# Transit Peptides From Photosynthesis-Related Proteins Mediate Import of a Marker Protein Into Different Plastid Types and Within Different Species

**DOI:** 10.3389/fpls.2020.560701

**Published:** 2020-09-25

**Authors:** Álvaro Eseverri, Can Baysal, Vicente Medina, Teresa Capell, Paul Christou, Luis M. Rubio, Elena Caro

**Affiliations:** ^1^Centre for Plant Biotechnology and Genomics, Universidad Politécnica de Madrid (UPM) - Instituto Nacional de Investigación y Tecnología Agraria y Alimentaria (INIA), Pozuelo de Alarcón, Spain; ^2^Departamento de Biotecnología-Biología Vegetal, Escuela Técnica Superior de Ingeniería Agronómica, Alimentaria y de Biosistemas, Universidad Politécnica de Madrid, Madrid, Spain; ^3^Department of Plant Production and Forestry Science, University of Lleida-Agrotecnio Center, Lleida, Spain; ^4^ICREA, Catalan Institute for Research and Advanced Studies, Barcelona, Spain

**Keywords:** plastid targeting, recombinant protein, plastid import, transit peptide, rice, crops, synthetic biology, biotechnology

## Abstract

Nucleus-encoded plastid proteins are synthesized as precursors with N-terminal targeting signals called transit peptides (TPs), which mediate interactions with the translocon complexes at the outer (TOC) and inner (TIC) plastid membranes. These complexes exist in multiple isoforms in higher plants and show differential specificity and tissue abundance. While some show specificity for photosynthesis-related precursor proteins, others distinctly recognize nonphotosynthetic and housekeeping precursor proteins. Here we used TPs from four *Arabidopsis thaliana* proteins, three related to photosynthesis (chlorophyll a/b binding protein, Rubisco activase) and photo-protection (tocopherol cyclase) and one involved in the assimilation of ammonium into amino-acids, and whose expression is most abundant in the root (ferredoxin dependent glutamate synthase 2), to determine whether they were able to mediate import of a nuclear-encoded marker protein into plastids of different tissues of a dicot and a monocot species. In *A. thaliana*, import and processing efficiency was high in all cases, while TP from the rice Rubisco small chain 1, drove very low import in Arabidopsis tissues. Noteworthy, our results show that Arabidopsis photosynthesis TPs also mediate plastid import in rice callus, and in leaf and root tissues with almost a 100% efficiency, providing new biotechnological tools for crop improvement strategies based on recombinant protein accumulation in plastids by the expression of nuclear-encoded transgenes.

## Introduction

Plastids are double-membrane organelles found within plants and algae cells. Phylogenetic analyses show that plastids originated from endosymbiosis of a cyanobacterial ancestor (Yoon et al., 2004). In higher plants, all plastids derive from undifferentiated pro-plastids that develop into different morphological and functional types in a developmental and tissue-specific manner and which have distinct functions mediated by different proteomes (Kleffmann et al., 2004; Siddique et al., 2006; Daher et al., 2010).

Plastid genomes of land plants have suffered huge reduction by transferring genes to the nuclear genome (Kleine et al., 2009). Such transfer underscores the importance of differentially expressing nuclear genes encoding plastid proteins according to tissue and developmental stage (Kleffmann et al., 2004) and the regulation of the import of proteins from the cytosol (Jarvis and López-Juez, 2013; Chu and Li, 2018; Chu et al., 2020), as these processes ultimately determine plastid biogenesis and plant development. Nuclear-encoded plastid-targeted proteins are synthesized as precursors containing an N-terminal targeting sequence called the transit peptide (TP) (Dobberstein et al., 1977; Chua and Schmidt, 1978; Kleffmann et al., 2004). TPs bind the targeting receptors associated with the translocons at the outer/inner envelope membrane of plastids (TOC and TIC, respectively) and direct the import of precursor proteins across the organellar double membrane (reviewed in Chotewutmontri et al., 2017). After precursor translocation into the stroma, the TP is cleaved off by the Stromal Processing Peptidase (SPP) and the mature protein folds into its native conformation and stays within the stroma or continues its journey to the thylakoids (Richter and Lamppa, 2002). TPs are necessary and sufficient for protein import into plastids; the removal of the gene part coding for it renders a protein that remains in the cytosol, while the addition of the TP to a nonplastid protein can direct it to the organelle (Bruce, 2001). The length of TPs is very heterogeneous, ranging from 20 to 150 amino acids, depending on the position of the processing site by the SPP (Balsera et al., 2009). TP primary sequence alignments have been used to identify conserved motifs responsible for specific import, but their conservation, amino acid composition and organization are very reduced (Bruce, 2000). A semi-conserved FGLK motif was found in several transit peptides (Karlin-Neumann and Tobin, 1986) and shown to interact with the translocation apparatus (Wienk et al., 2000; Schleiff et al., 2002; Holbrook et al., 2016). Other motifs have been identified as relevant, like the FP-RK, whose inclusion in the nonfunctional *Chlamydomonas reinhardtii* RbcS allowed it to deliver proteins into Arabidopsis chloroplasts (Razzak et al., 2017), and an abundance of Pro residues, shown to mediate efficient translocation of proteins containing transmembrane domains or proteins prone to aggregation (Lee et al., 2017). It also seems that TPs adopt alpha-helical structures in membrane-mimetic environments and that this structure might play a role in TP recognition (Bruce, 2001).

The general import pathway seems to be very conserved, as evidenced by the fact that most TOC/TIC components are maintained in all land plant species (Tello-Ruiz et al., 2016). Their expression is highly regulated. In *Arabidopsis thaliana*, the receptors for the initial pre-protein binding, atToc59 and atToc33 are highly expressed in green tissues, while atToc132/120 and atToc34 are uniformly expressed across all tissues (reviewed in Chu and Li, 2018). Arabidopsis and rice TIC/TOC systems are very conserved in terms of members of the complex and their specific expression patterns (**Supplementary Figure 1**).

Recent data suggest that the availability of TOC isoforms that bind with different specificity to each transit peptide can determine the import of precursor proteins (Jarvis and López-Juez, 2013; Dutta et al., 2014; Chu and Li, 2018; Chu et al., 2020). *In vitro* binding assays showed that atToc159 binds preferentially to transit peptides of photosynthetic proteins and atToc132 binds transit peptides of nonphotosynthetic proteins (Ivanova et al., 2004; Smith et al., 2004; Inoue et al., 2010). Swapping of TPs among proteins confirmed their role in the determination of plastid-type import selectivity (Wan et al., 1996; Yan et al., 2006). The precursor-specificity of the pathway seems to be restricted to the TOC, since TIC components were found to associate equally with all kinds of plastid imported proteins (Chen et al., 2002; Kovacheva et al., 2005; Jarvis, 2008).

Detailed characterization of different TPs, and their specific plastid-type import preference, is of the utmost importance to engineer useful traits determined by nuclear-encoded recombinant plastid proteins. Here we show that the TPs of three Arabidopsis proteins related to photosynthetic and photo-protection processes direct highly efficient import of a recombinant reporter protein to chloroplasts and leucoplasts. These TPs also show 100% import efficiency in rice callus and in the shoot and root tissues of rice plants, suggesting that translocon components mediating import of the reporter protein are distributed equally in these tissues or that the TPs bind promiscuously to predominant TOC/TIC isoforms. These findings will lead to the development of synthetic biology tools for direct use in crop biotechnology.

## Materials and Methods

### Molecular Biology

All constructs used in this work were generated using MoClo (Weber et al., 2011; Werner et al., 2012) and its adaptation for the direct DNA transfer to rice (Baysal et al., 2020). The design of primers to generate Level 0 plasmids was performed manually following the rules described in Weber et al. (2011). When DNA fragments contained internal restriction sites for either *Bsa*I or *Bpi*I, the fragment was domesticated using internal primers to eliminate the site. The design of domestication primers was performed using the Domesticator Tool (https://gbcloning.upv.es/do/domestication/), subsequently modifying the tails of the primers to add a *Bpi*I restriction site. Modular DNA fragments were amplified by PCR using Phusion Hot Start II DNA Polymerase (Thermo Fisher Scientific, Waltham, MA, USA) and the primers and templates described in **Supplementary Data 1**.

MoClo restriction-ligations were set up in a final volume of 20 μl with a 2:1 molar ratio of insert:acceptor vector, 5U of the required restriction enzyme – *Bpi*I (Thermo Fisher Scientific) for the generation of Level 0 and Level 2 plasmids, and *Bs*aI-HFv2 (NEB, Ipswich, MA, USA) for the generation of Level 1 plasmids – 4.5U of T4 Ligase (Promega, Madison, WI, USA), 1.5 μl of Ligase 10x Buffer and 1.5 μl of 10x BSA (GE Healthcare, Chicago, IL, USA). The reactions were incubated in a thermocycler with the following program: 20 s at 37°C, 26 cycles of 3 min at 37°C plus 4 min at 16°C, and 5 min at 50°C, 5 min at 80°C and hold at 16°C.

The reaction mixes were then added to chemically competent cells of *Escherichia coli* TOP10 (Thermo Fisher Scientific), which were then incubated in LB solid medium containing 20 μg/ml X-Gal (Duchefa Biochemie, Haarlem, Netherlands), 1 mM IPTG (Sigma-Aldrich, Darmstadt, Germany), and the corresponding antibiotic, 50 μg/ml spectinomycin (Sigma-Aldrich) for Level 0, 100 μg/ml carbenicillin (Formedium, Norfolk, UK) for Level 1, or 50 μg/ml kanamycin (Formedium) for Level 2 constructs. White colonies were selected for plasmid DNA extraction using the QIAprep Spin Miniprep Kit (Qiagen, Hilden, Germany). The fidelity of all plasmids was verified by Sanger sequencing (Eurofins Genomics, Ebersberg, Germany).

The transcriptional units and multigene constructs generated in this work are listed in **Supplementary Data 2** and **3**, respectively.

### Plant Growth

*Arabidopsis thaliana* Col-0 seeds were plated in MS medium pH 5.7 (Murashige and Skoog, 1962) (Mod.No.1B, Duchefa) with 1% (w/v) sucrose (Duchefa) and 1% (w/v) plant agar (Duchefa). Seedlings were grown in an environmentally controlled chamber under 16/8 h light/dark conditions at 22°C and 65% relative humidity.

Rice seeds (*Oryza sativa* L. cv. EYI 105) were sown into a 3:1 mixture of soil (Floragard, Oldenburg, Germany) and vermiculite in ⌀ 13 cm pots and grown for 9 days after germination in an environmentally controlled chamber (Conviron, Winnipeg, Canada) under 16/8 h light/dark conditions at 24°C and 70% relative humidity.

### Protoplast Isolation

Isolation of protoplast from Arabidopsis seedlings was performed as described in Yoo et al. (2007) and González-García et al. (2020) with modifications. Aerial parts and roots of ca. 100 seedlings were separated and cut into 0.5–1.0 mm strips using a razor blade. The strips were transferred to different Erlenmeyer flasks containing 10 ml of enzyme solution (1.25% (w/v) cellulase R10 (Duchefa), 0.3% (w/v) macerozyme R10 (Duchefa), 0.4 M D-mannitol, 20 mM MES at pH 5.7, 20 mM KCl, 10 mM CaCl_2_ and 0.1% (w/v) BSA) and incubated in the dark for 3 h (aerial part) or 1 h and 45 min (roots) at RT with 80 rpm shaking to allow digestion of cell wall material. An equal amount of W5 solution (154 mM NaCl, 125 mM CaCl_2_, 5 mM KCl and 2 mM MES at pH 5.7) was added to stop the digestion. Protoplasts were released by filtering through 40 μm nylon cell strainers and pelleted by centrifugation at 200 × *g* for 2 min (aerial part) or 500 × *g* for 10 min (roots) at room temperature. Protoplast were resuspended in 2 ml MMG solution (0.4 M D-mannitol, 15 mM MgCl_2_ and 4 mM MES at pH 5.7) and kept on ice.

Isolation of protoplast from rice seedlings was performed as described in Zhang et al. (2011) and Page et al. (2019).

### Transient Expression in Protoplasts

Transformation of protoplasts was carried out by DNA-PEG-calcium transfection as described in Yoo et al. (2007) and Page et al. (2019). For each transformation, 10 μl of plasmid (5 to 10 μg DNA) and 100 μl of protoplasts were combined with 110 μl of a freshly-prepared solution containing 40% (w/v) PEG 4000 (Merk, Darmstadt, Germany), 0.2 M D-mannitol and 0.1 M CaCl_2_, and incubated in the dark at room temperature for 15–25 min. Negative control samples, replacing plasmid by 10 μl H_2_O, were carried out. 440 μl of W5 solution was slowly added to complete transformations. Protoplasts were pelleted by centrifugation, resuspended in 375 μl of WI solution (0.5 M D-mannitol, 20 mM KCl and 4 mM MES at pH 5.7), transferred to 96‐well microplates (125 μl per well), and incubated at room temperature on the laboratory bench.

### Transformation of Rice Callus and Regeneration of Transgenic Rice Plants

The pUC57 based transcriptional units containing eGFP fused to the individual chloroplast transit peptides, namely *At*mCAB6_TP_, *At*mTOCC_TP_ and *At*mRCA_TP_, (**Supplementary Data 2B**) were introduced separately into rice nature-embryo derived callus rice embryos, together with the *hpt* gene for selection as described in Christou et al. (1991) and Sudhakar et al. (1998). A minimum of two representative independent callus lines and the corresponding regenerated plants for each construct were selected for in depth analyses.

### Analysis of Protein Expression

Arabidopsis and rice transformed protoplasts were recovered from 96-well plates by gentle pipetting, transferred to 1.5 ml Eppendorf tubes and pelleted by centrifugation at 100 × *g* for 2 min (Arabidopsis mesophyll cells), 500 × *g* for 10 min (Arabidopsis root cells) or 300 × *g* for 6 min (rice cells) at room temperature. Supernatants were discarded and pellets were shock frozen in liquid N_2_. Protoplasts were re-suspended in 2x Laemmli buffer (125 mM Tris-HCl pH 6.8, 4% (w/v) SDS, 20% (v/v) glycerol, 10% (v/v) β-mercaptoethanol, 0.005% (w/v) bromophenol blue) and subsequently boiled for 10 min, cooled down and centrifuged at 14,000 × *g* for 3 min at 4°C to obtain total protein extracts.

Rice callus was harvested and placed into 1.5 ml Eppendorf tubes. Rice leaf and root tissue samples were excised from plantlets when transferring them to soil and harvested into 2-ml screw-cap tubes containing five 3-mm diameter glass beads, weighted, immediately frozen in liquid N_2_, and then stored at -80°C until further use. Callus tissue was homogenized in the presence of ice-cold protein extraction buffer composed of 100 mM HEPES pH 7.2, 1 mM MgCl_2_, 10 mM DTT, 10% (w/v) glycerol, and 0.5% (v/v) PIC (Sigma-Aldrich) with the help of a stainless-steel pestle, and subsequently centrifuged at 14,000 × *g* for 3 min at 4°C to obtain soluble protein extracts. Rice leaf and root tissue samples were ground to a fine powder with the help of a plastic pestle and a BeadBug homogenizer (Benchmark Scientific Inc, Sayreville, NJ, USA), mixed with 4 volumes of 2× Laemmli buffer, boiled for 10 min, cooled down and centrifuged at 14,000 × *g* for 3 min at 4°C to obtain total protein extracts.

SDS-PAGE and immunoblot analysis were performed by standard methods. Samples processed with protein extraction buffer were resuspended in Laemmli sample buffer 2×, where crude extracts were used directly. A commercially available antibody against GFP (11814460001, Roche, Basel, Germany) diluted 1:3,000 was used in combination with a secondary horseradish peroxidase-conjugated anti-mouse IgG diluted 1:10,000 (AS11 1772, Agrisera AB, Vännäs, Sweden).

Immunoblot membranes were developed and then visualized in an iBright FL1000 Imaging System (Thermo Fisher Scientific) using chemiluminescence mode. *Smart exposure* setting was used for all blots except when performing long exposures. iBright Analysis Cloud Service (Thermo Fisher Scientific) was used for image processing, which was equally applied across the entire image including control lines, and for determination of protein band signal intensities. Import efficiency was defined as the percentage of the processed faster-migrating protein form relative to the total amount of expressed protein in each line. Analyses were performed in at least three biological replicates.

### Microscopy

Protoplast from Arabidopsis and rice cells were placed on a Neubauer counting chamber (Marienfeld-Superior, Lauda-Königshofen, Germany) and imaged using a Zeiss AxioPhot Microscope (Zeiss, Oberkochen, Germany) equipped with a Plan-Neofluar 40X/0.75 objective, coupled to a color CCD Leica DFC 300FX camera (Leica, Wetzlar, Germany) and LAS software. Bright Field images were processed with LAS software and assembled using Illustrator software (Adobe, San José, CA, USA).

For confocal microscopy analyses, protoplasts were imaged using a Zeiss LSM880 confocal laser scanning microscope (Zeiss) equipped with a Plan-Apochromat 40X/1.2 water-immersion or Plan-Apochromat 63X/1.2 oil-immersion objective and ZEN 2.6 Black software (Zeiss). The excitation laser lines wavelength/emission bands used with PMT detectors were as follows: GFP (488 nm/493 to 556 nm), mCherry (561 nm/595 to 640 nm), and chlorophyll autofluorescence (633 nm/647 to 721 nm). Super-resolution (AS-SR) imaging was performed with an Airyscan detection unit, sequentially, with excitation as described above and with emission band pass filters as follows: GFP (488 nm/BP 420-480 + BP 495-550), mCherry (BP 420-480 + BP 495-620), and chlorophyll auto-fluorescence (633 nm/BP 570-620 + LP 645). ZEN 2.6 Black software was used to process the AS-SR acquired datasets with the automatically-determined Airyscan Filtering. Images were post-processed with ZEN 2.6 Blue, where Z axis maximum intensity projections were performed, and assembled using Illustrator software (Adobe).

Rice callus was imaged using a Fluorescent Stereo Microscope Leica MZ10 F (Leica) equipped with a Plan-Apochromat 1.0×/0.125 objective and Leica Application Suite (LAS) v4.3 software (Leica). A GFP Plus filter was set with the following parameters to detect eGFP: excitation wavelength 460–500 nm/emission 510 nm. Images were processed with LAS software and assembled using Illustrator software (Adobe).

For electron microscopy immune detection, pieces of rice callus (1 mm^3^) or leaf tissue (1 × 10 mm) or root (10 mm) were fixed with 1% glutaraldehyde and 1% paraformaldehyde in 0.1 M sodium phosphate buffer (pH 7.2) for 16–24 h at 4°C. To enhance the penetration of fixative, samples were initially subjected to a light vacuum until the tissue pieces sank. Once fixed, pieces were washed three times (10 min) with the same buffer and dehydrated in an ethanol series (30%–100%) before embedding in Lowicryl K4 M resin (Polysciences, Hirschberg an der Bergstrasse, Germany) in a cold chamber at −20 to −35°C and inducing polymerization by exposure to ultraviolet light.

Semithin (2 µm) and ultrathin (70–90 nm) sections were prepared using a Reichert–Jung ultra-cut E cryotome (Leica). The semithin sections were stained with Richardson’s blue, covered with a drop of DPX slide mounting medium and a coverslip, and observed under a DM4000B microscope (Leica). Images were captured using a DFC300 FX 1.4-MP digital color camera equipped with LAS v3.8 (Leica). The ultrathin sections were mounted on Formvar carbon-coated gold grids (200 mesh) and incubated for 30 min in blocking buffer for polyclonal antibodies (200 mM Tris–HCl at pH 7.4, 1% Tween-20, 0.1% gelatin, 1% BSA) or monoclonal antibodies (10 mM Tris–HCl at pH 7.4, 0.9% NaCl, 0.05% PEG 20,000, 3% BSA). The grids were then washed in distilled water and incubated overnight at 4°C with primary polyclonal anti-eGFP antibody PA5-22688 (Thermo Fisher Scientific) diluted 1:200 in blocking buffer, or primary monoclonal anti-eGFP antibody 11814460001 (Sigma-Aldrich) diluted 1:250 in blocking buffer. After washing in distilled water, followed by a further 30-min incubation in the appropriate blocking buffer and another wash, the grids were incubated at room temperature for 1 h with the 15-nm gold-conjugated secondary antibody diluted 1:20 in the appropriate blocking buffer: goat-anti-rabbit IgG for the polyclonal antibody, or EM-grade goat-anti-mouse IgG for the monoclonal antibody (Electron Microscopy Sciences, Hatfield, PA, USA). Finally, the grids were contrasted with 1% uranyl acetate in water (20 min) and Reynold’s lead citrate (2 min) before observation in Jeol Jem-1010 Transmission Electron Microscope (Jeol Ltd., Tokyo, Japan). A minimum of two grids per treatment and sample were analyzed.

Leaf tissue samples (1 × 10 mm) were fixed with 2% paraformaldehyde in 0.1 M sodium phosphate buffer (pH 7.2) and cut into semi-thin sections (30–40 lm) using a CM3050S Research Cryostat (Leica Microsystems, Wetzlar, Germany). The sections were collected on standard glass microscope slides pre-coated with poly-L-lysine and images were captured using an FV1000 laser scanning confocal microscope (Olympus, Hamburg, Germany) with illumination at 488 nm (excitation wavelength of eGFP, multiline argon laser).

## Results

### Import Plastid-Type Specificity

**Figure 1:**
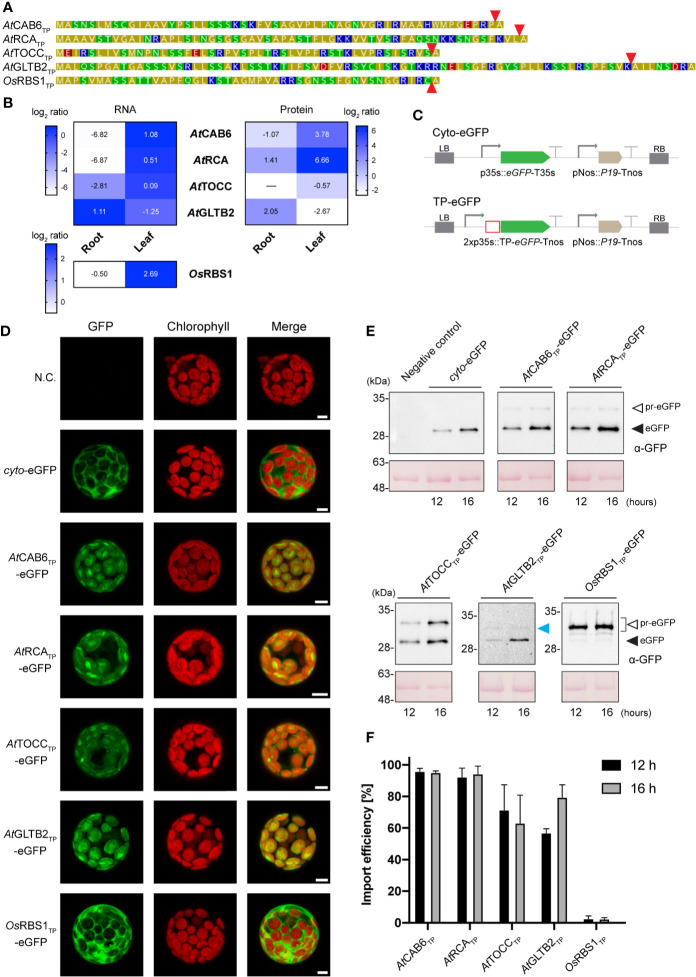
*In vivo* chloroplast targeting of TP-eGFP fusion proteins in Arabidopsis leaf protoplasts. **(A)** Amino acid sequence of transit peptides (TPs) from *A. thaliana* and *O. sativa*. Coloring according to amino acid polarity as follows: Yellow: Non-polar; Green: Polar, uncharged; Red: Polar, acidic; Blue: Polar, basic. Red arrows indicate predicted Stromal Processing Peptidase (SPP) cleavage site. **(B)** Gene expression and protein levels of corresponding TP genes and mature proteins in *A. thaliana* and *O. sativa*. Gene expression data was retrieved from the Plant eFP browser, showing log_2_ of fold change from microarray transcriptomics (Waese et al., 2017). Protein data was retrieved from AtProteome (Baerenfaller et al., 2008). Heatmap shows log_2_ ratio from shotgun proteomics. **(C)** Schematic representation of the constructs used in *A. thaliana* transient expression assays leading to eGFP expression and localization. **(D)** Confocal laser scanning microscopy images of *A. thaliana* leaf protoplast expressing indicated TP-eGFP fusions. N.C. corresponds to “Negative Control”, nontransformed protoplasts. The columns show individual signals for eGFP (green, on the left) and chlorophyll autofluorescence (red, in the center). On the right, overlap of both signals (merge). Scale bars = 5 µm. **(E)** Western-blot analysis of import experiments. Protoplast isolated from *A. thaliana* leaves were transformed with indicated TP-eGFP fusions and total protein extracts were analyzed by Western blotting using anti-GFP antibody. The experiments were performed in three biological replicates and representative data is shown. White arrows (pr-eGFP) indicate precursor form; black arrows (eGFP) indicate, processed form; blue arrow indicates partially processed form. **(F)** Quantification of import efficiency of TPs into Arabidopsis chloroplast. Import efficiency was defined as the percentage of the processed faster-migrating protein form relative to the total amount of expressed eGFP protein in each line. Data represent means (n = 3) with SD.

We had previously reported the minimal versions of thirteen TPs from different plant origins that were able to direct bacterial recombinant proteins to tobacco chloroplasts (Eseverri et al., 2020). To test plastid specificity, here we selected three of these TPs from Arabidopsis proteins directly involved in photosynthesis (CAB6, chlorophyll a/b binding protein; RCA, Rubisco activase) or photo-protection (TOCC, tocopherol cyclase) and one involved in the assimilation of ammonium into amino-acids (GLTB2, ferredoxin dependent glutamate synthase 2) (**Figure 1A**; **Supplementary Figure 2**). CAB6, RCA and TOCC are highly expressed in green tissue (**Figure 1B**, left panel), and accumulate preferentially in leaves (**Figure 1B**, right panel), while GLTB2 expression and protein accumulation are most abundant in roots, as evidenced by data from the Plant eFP browser (Waese et al., 2017) and AtProteome (Baerenfaller et al., 2008) databases. It is interesting to note that TOCC was also expressed at low levels in roots, consistent with recent studies that suggest a role of tocopherols in the adaptation to drought (Cela et al., 2011) and heavy metals (Collin et al., 2008).

**Figure 2:**
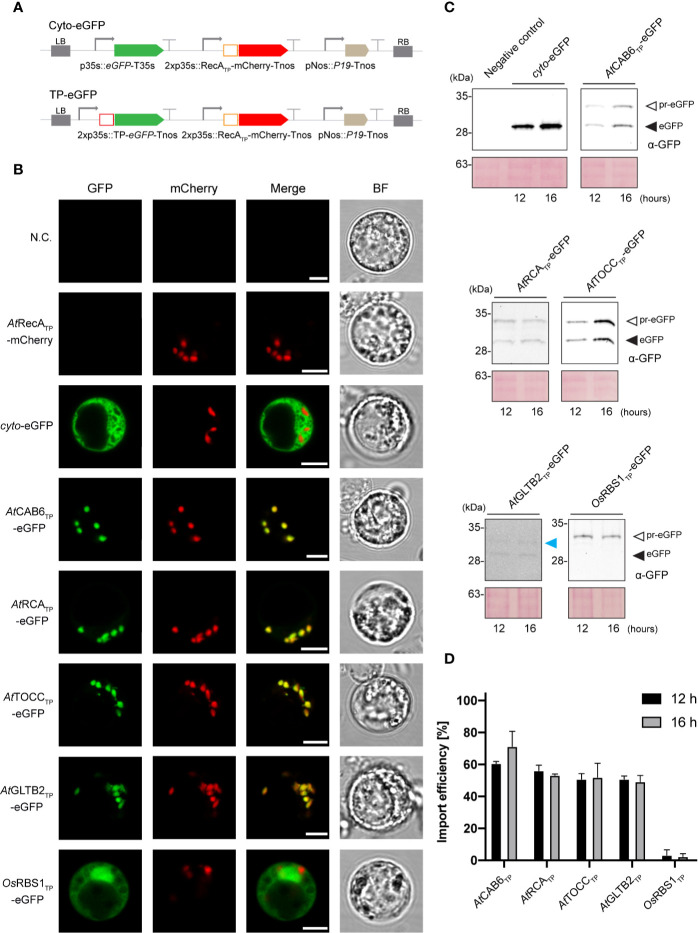
*In vivo* leucoplast targeting of TP-eGFP fusion proteins in Arabidopsis root protoplasts. **(A)** Schematic representation of the constructs used in *A. thaliana* transient expression assays leading to eGFP expression and localization. **(B)** Confocal laser scanning microscopy images of *A. thaliana* root protoplast expressing indicated TP-eGFP fusions. N.C. corresponds to “Negative Control”, nontransformed protoplasts. The four columns show individual signals for eGFP (green, on the far left), mCherry (red, on the left) and transmitted light (BF, on the far right). On the right, overlap of eGFP and mCherry signals (merge). Scale bars = 5 µm. **(C)** Western-blot analysis of import experiments. Protoplast isolated from *A. thaliana* roots were transformed with indicated TP-eGFP fusions and total protein extracts were analyzed by Western blotting using anti-GFP antibody. The experiments were performed in three biological replicates and representative data is shown. White arrows (pr-eGFP) indicate precursor form; black arrows (eGFP) indicate, processed form; blue arrow indicates partially processed form. **(D)** Quantification of import efficiency of TPs into Arabidopsis root plastids. Import efficiency was defined as the percentage of the processed faster-migrating protein form relative to the total amount of expressed eGFP protein in each line. Data represent means (n = 3) with SD, except GLTB2 and RBS1 TPs, where n=2.

Constructs in which these four TPs were fused to the reporter protein eGFP were generated, together with the rice Rubisco small chain 1 (RBS1) TP, which is highly expressed in leaves, to analyze import mediated by a TP from a different species (**Figures 1A, B**), and a eGFP cytosolic form for control of the matured form size (**Figures 1C, 2A**), and used to transform protoplasts obtained from Arabidopsis mesophyll (**Figures 1D, E**; **Supplementary Figure 3**) and root cells (**Figures 2B, C**; **Supplementary Figure 3**). Chloroplasts were easily identified in protoplasts of mesophyll cells by their red auto-fluorescence. The plastid-targeted eGFP coincided with chlorophyll fluorescence for all Arabidopsis TPs, whereas for the rice RBS1 TP and the cytosolic control, eGFP fluorescence was localized outside the chloroplasts (**Figure 1D**). These results are consistent with our previous observations in tobacco (Eseverri et al., 2020), and demonstrate that the selected TP sequences contain all necessary motifs to correctly direct import of nuclear-encoded recombinant proteins into chloroplasts.

Expression of the Arabidopsis TP-eGFP fusions in protoplasts obtained from Arabidopsis root cells rendered a fluorescence overlapping with that produced by the expression of RecA-mCherry, an established root plastid marker (Nakata et al., 2018) and different to the one observed for the rice RBS1 TP and the cytosolic control (**Figure 2B**). This result suggests that the Arabidopsis TPs also mediate successful import of eGFP into root leucoplasts.

SDS-PAGE and immunoblot analysis of protein extracts from Arabidopsis mesophyll (**Figure 1E**) and root protoplasts (**Figure 2C**) taken at two different time points (12 and 16 h after transformation) made the evaluation of the dynamics of protein import possible. The results show that although some increase in total protein abundance could be observed after 16 h, import efficiency is mostly stable and not dependent on time. The immunoblots showed the presence of lower mobility bands, in some cases, with size corresponding to the pre-protein (pr-eGFP), in others, smaller, which could correspond to processing within the TP, and a higher mobility band with size consistent with the processed eGFP protein after SPP cleavage during import. The ratio of processed to total protein provides an estimate of import efficiency for each TP in leaf and root plastids (**Figures 1F** and **2D**, respectively). Although some preference was noted for chloroplast import in the case of CAB6 (96% and 95%, at 12 h and 16 h respectively) and RCA TPs (92% and 94%), both still showed relatively high efficiency in the import to root leucoplasts (61%–70% and 56%–54%, respectively). Conversely, TOCC and GLTB2 TPs showed medium import efficiency into chloroplasts (71%–63% and 57%–79%), and also a bit lower in leucoplasts (51%–52% and 51%–49%, respectively). This is a surprising result for GLTB2, given that its actual function is performed in the root tissue where it is preferentially expressed. The rice RBS1 TP was unable to correctly target eGFP into chloroplasts (2%–2%, at 12 h and 16 h respectively) or leucoplasts (3%–2%) of Arabidopsis, accumulating as a pre-protein (**Figures 1E, 2C**), and suggesting a species-specific import mechanism. For full data and statistics, please see **Supplementary Table 1**.

### Conservation of the Import Mechanism

**Figure 3:**
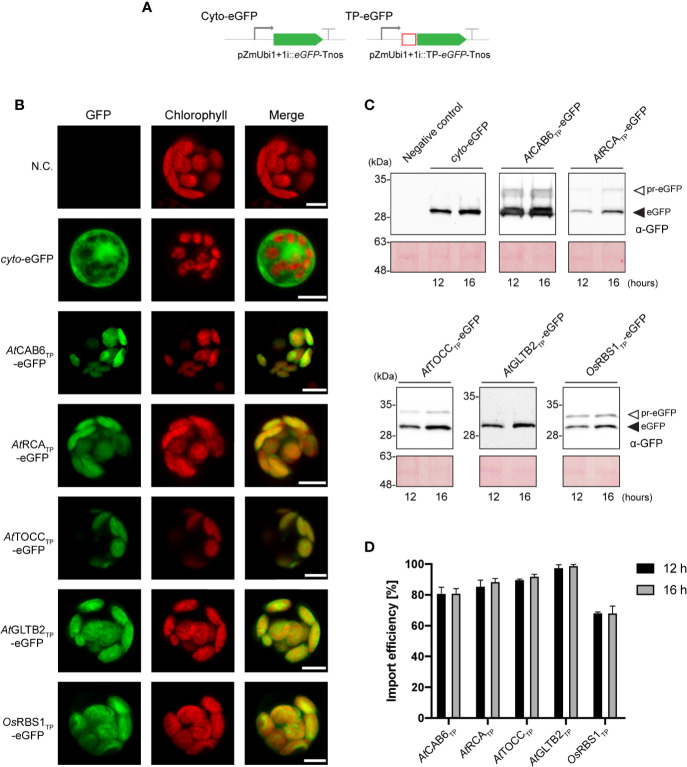
Arabidopsis transit peptides (TPs) are able to direct eGFP into rice chloroplast. **(A)** Schematic representation of the constructs used in *O. sativa* transient expression assays leading to eGFP expression and localization. **(B)** Confocal laser scanning microscopy images of rice protoplast expressing indicated TP-eGFP fusions. N.C. corresponds to “Negative Control”, nontransformed protoplasts. The three columns show individual signals for eGFP (green, on the left), chlorophyll autofluorescence (red, in the center) and overlap of both signals (right). Scale bars = 5 µm. (**C)** Western-blot analysis of import experiments. Protoplast isolated from rice were transformed with indicated TP-eGFP fusions and total protein extracts were analyzed by western blotting using anti-GFP antibody. The experiments were performed in three biological replicates and representative data is shown. White arrows (pr-eGFP) indicate precursor form; black arrows (eGFP) indicate, processed form. **(D)** Quantification of import efficiency of TPs into rice chloroplast. Import efficiency was defined as the percentage of the processed faster-migrating protein form relative to the total amount of expressed eGFP protein in each line. Data represent means (n = 3) with SD.

The identification of Arabidopsis TPs that mediate effective plastid import of recombinant proteins in both, Arabidopsis (CAB6, RCA, TOCC, GLTB2, this work) and tobacco (CAB6, RCA and TOCC, Eseverri et al. (2020), and of a rice TP that is not able to mediate import in Arabidopsis (RBS1, this work) provided a basis for further investigation. Therefore, we deepened our study in the extent of conservation of plastid translocation mechanisms between Arabidopsis and the staple species, rice. To this end, eGFP constructs analogous to those we described above were designed for expression in monocot cells (**Figure 3A**) and used to transform protoplasts of cells from rice sheath and stem (**Supplementary Figure 3**). The plastid-targeted eGFP signal coincided with chlorophyll fluorescence in all cases, in contrast to that of the cytosolic eGFP (**Figure 3B**), confirming that all the selected TPs were, as opposed to previous results in Arabidopsis, effective in rice cell protoplasts.

SDS-PAGE and immunoblots analysis of transformed rice protoplast extracts showed a lower mobility band corresponding to pre-eGFP and a higher mobility band corresponding to processed eGFP (**Figure 3C**). Interestingly, import efficiencies observed were, in the case of CAB6 (80%–81%, at 12 h and 16 h respectively) and RCA (85%–88%) TPs, slightly lower than those observed for Arabidopsis chloroplasts, and higher in the case of TOCC (89%–92%) and GLTB2 (97%–99%) (**Figure 3D**). The rice endogenous RBS1 TP was now able to direct eGFP into chloroplasts, but, surprisingly, less efficiently (68%–68%, at 12 h and 16 h respectively) than its counterparts from Arabidopsis. For full data and statistics, please see **Supplementary Table 1**.

### Arabidopsis TPs Use as Tools for Plastid Import in Whole Rice Plants

**Figure 4:**
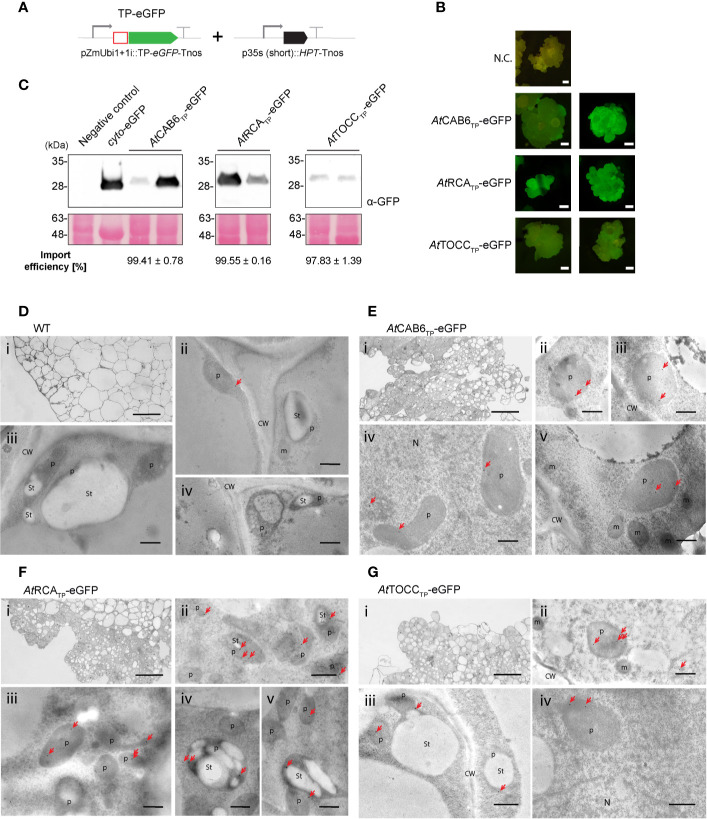
Stably transformed rice callus lines exhibit eGFP targeting into rice pro-plastids by Arabidopsis transit peptides (TPs). **(A)** Schematic representation of the constructs used for stable transformation of *O. sativa*, leading to eGFP expression and localization. **(B)** Stereomicroscope fluorescence images from two independent transformed callus lines expressing *At*CAB6_TP_-eGFP, *At*RCA_TP_-eGFP, or *At*TOCC_TP_-eGFP. N.C. corresponds to “Negative Control”, a transformed callus line expressing only the HPT resistance cassette. Scale bars = 1 mm. **(C)** Western blot analysis of callus soluble protein extracts from two independent transformed rice lines with the indicated constructs incubated with anti-GFP antibody. eGFP produced in *N. benthamiana* was used as a positive size control. Import efficiency was defined as the percentage of the processed faster-migrating protein form relative to the total amount of expressed eGFP protein. Data shown are means ± SD of three independent extractions. **(D-G)** Immunogold labeling of eGFP in proplastids of rice callus cells using a GFP-specific monoclonal antibody (diluted 1:250). **(D)** Wild-type cells. **(E)** *At*CAB6_TP_-eGFP. **(F)** *At*RCA_TP_-eGFP. **(G)** *At*TOCC_TP_-eGFP. i: Light microscopy (LM) image of studied section; ii-v: Transmission electron microscope (TEM) images. (CW, cell wall; m, mitochondria; N, nucleus; p, plastid like; St, starch; bars i = 50 µm, ii–v = 500 nm; gold particle size = 15 nm).

In light of the previous results supporting the efficiency of Arabidopsis TPs in rice green tissue, we generated transgenic rice lines in which rice embryos were co-transformed with a plasmid carrying a hygromycin selection cassette and the CAB6, RCA and TOCC TPs fused to the N terminus of eGFP (**Figure 4A**). Several independent embryogenic callus lines were recovered from each combination of plasmids in which eGFP was expressed to such levels as to be visible under the stereomicroscope (**Figure 4B**). Lines recovered after transformation with the construct containing the TOCC TP had lower expression levels compared to CAB6 or RCA TPs (as evidenced by fluorescence emission and protein accumulation). Inmmunoblot analyses confirmed in all three cases, that only the fully cleaved version of eGFP was detected in callus proplastids, indicating an almost 100% import efficiency (**Figure 4C**, **Supplementary Table 1**).

Localization of eGFP within callus proplastids was confirmed using immunogold labeling and electron microscopy. When using a monoclonal antibody against eGFP, callus lines showed very low levels of labeling, but very specific association of particles with the interior of plastids (**Figures 4D–G**). Labeling using polyclonal antibodies against eGFP produced significant nonspecific labeling, also visible in the wild-type callus, especially in nuclei and cytoplasm, but also higher signal density could still be observed within plastids (**Supplementary Figure 4**), consistent with our earlier results (**Figures 4D–G**). An estimate quantification of the immunogold labeling results can be found in **Supplementary Table 2**.

**Figure 5:**
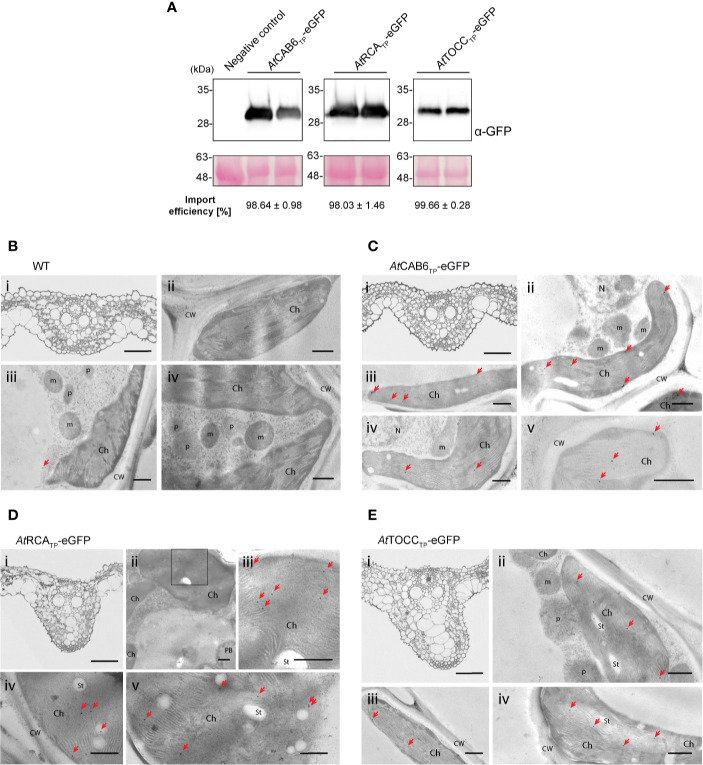
Regenerated rice plants show eGFP targeting into chloroplast driven by Arabidopsis transit peptides (TPs). **(A)** Western blot analysis of leaf total protein extracts from two plants regenerated from the same transformed callus line expressing the indicated constructs above, incubated with anti-GFP antibody. Negative Control represents a wild-type plant. Import efficiency was defined as the percentage of the processed faster-migrating protein form relative to the total amount of expressed eGFP protein. Data shown are means ± SD of three independent extractions. **(B-E)** Immunogold labeling of eGFP in chloroplast of rice leaf cells using a GFP-specific monoclonal antibody (diluted 1:250). **(B)** Wild-type cells. **(C)** *At*CAB6_TP_-eGFP. **(D)** *At*RCA_TP_-eGFP. **(E)** *At*TOCC_TP_-eGFP. i: Light microscopy (LM) image of studied section; ii–v: Transmission electron microscope (TEM) images. (Ch, chloroplast; CW, cell wall; m, mitochondria; N, nucleus; p, plastid like organelle; St, starch; bars i = 50 µm, ii-v = 500 nm; gold particle size = 15 nm).

Whole transgenic rice plants were regenerated from the eGFP expressing transformed callus in order to study the Arabidopsis TPs import efficiency in different rice tissues. In leaves, immunoblot analysis revealed an almost 100% import efficiency for all three TPs (**Figure 5A**; **Supplementary Table 1**), much higher than the one observed in the protoplast transient expression system (**Figure 3**). Electron microscopy immunogold detection showed labeling of chloroplasts, easily identifiable by their thylakoid structures, both by monoclonal (**Figures 5B–E**) and polyclonal antibodies (**Supplementary Figure 4**). An estimate quantification of the immunogold labeling results can be found in **Supplementary Table 2**. Furthermore, confocal microscopy analysis showed co-localization of the eGFP signal with chlorophyll autofluorescence (**Supplementary Figure 5**), supporting correct plastid targeting mediated by the TPs in study.

**Figure 6:**
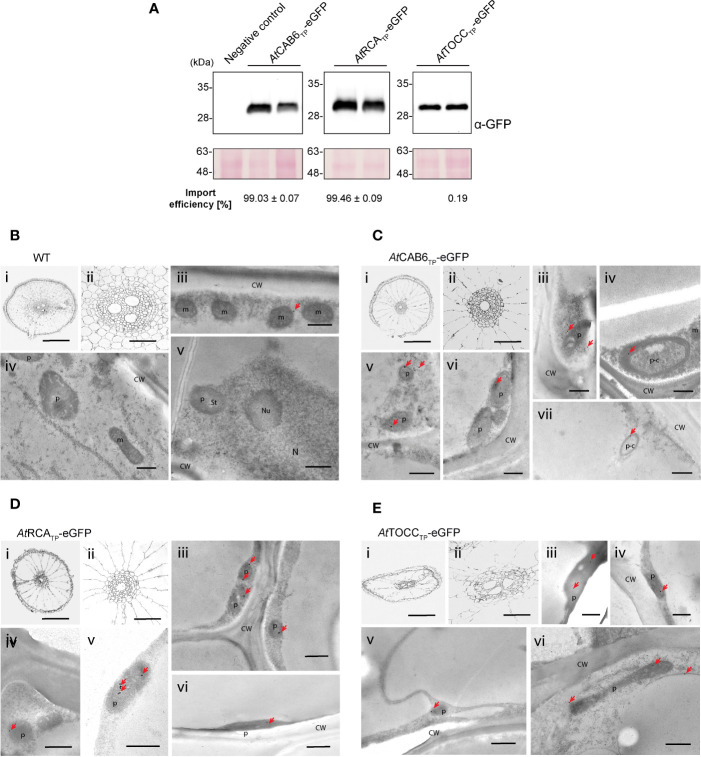
Roots from regenerated rice plants show eGFP targeting into leucoplasts driven by Arabidopsis transit peptides (TPs). **(A)** Western blot analysis of root total protein extracts from two plants regenerated from the same transformed callus line expressing the indicated constructs above, incubated with anti-GFP antibody. Negative Control represents a wild-type plant. Import efficiency was defined as the percentage of the processed faster-migrating protein form relative to the total amount of expressed eGFP protein. Data shown are means ± SD of three independent extractions. **(B-E)** Immunogold labeling of eGFP in plastids of rice root cells using a GFP-specific monoclonal antibody (diluted 1:250). **(B)** Wild-type cells. **(C)** *At*CAB6_TP_-eGFP. **(D)** *At*RCA_TP_-eGFP. **(E)** *At*TOCC_TP_-eGFP. i: Light microscopy (LM) image of studied section; ii: detail of i (central cylinder); iii–vii: Transmission electron microscope (TEM) images. (CW, cell wall; m, mitochondria; N, nucleus; Nu, nucleolus; p, plastid-like; p-c, plastid showing an isolated cytoplasmic region; bars i = 0.2 mm, ii = 50 µm, iii–vii = 500 nm; gold particle size = 15 nm).

Similar import efficiency was observed in roots of transgenic rice plants. A single band corresponding to mature eGFP was observed for the three TPs in their respective immunoblots, indicating again almost 100% import efficiency (**Figure 6A**; **Supplementary Table 1**). Immunogold labeling using monoclonal antibodies showed weaker signals than in leaves or callus, but the gold particles were localized within leucoplasts (**Figures 6B–E**). Polyclonal antibodies rendered nonspecific labeling (also visible in the wild-type roots, especially in the cytoplasm) but the highest signal density was observed within leucoplasts (**Supplementary Figure 4**). For an estimate quantification of the signal, see **Supplementary Table 2**.

## Discussion

Earlier studies have identified preprotein specificities of import (Fitzpatrick and Keegstra, 2001; Jarvis and López-Juez, 2013; Chu and Li, 2018; Chu et al., 2020). However, our limited knowledge on this subject still makes a difficult task trying to infer pre-protein recognition and translocation efficiencies from the simple analysis of TPs primary sequence and TP behavior needs to be confirmed experimentally.

Apart from a general enrichment in alanine, serine and uncharged amino acid residues, no consensus sequence motifs have been identified among TPs (Ivey et al., 2000; Zybailov et al., 2008). The TP sequences used here to direct the nuclear-encoded eGFP marker protein into plastids exhibit these characteristics, i.e. a prevalence in serine, alanine and uncharged residues (cysteine, threonine, tyrosine, asparagine and glutamine) compared to that of the mature protein (**Figure 1A**; **Supplementary Figure 6**).

Two motifs were identified in the TP of Arabidopsis RuBisCO small subunit that are responsible for its Toc159-dependent import pathway into chloroplasts (Lee et al., 2009). The first one, (DITSITSNGG), could not be found among the TPs in study here (**Figure 1A**). The second motif, consisting in a group of serine residues (SS), was present in CAB6, TOCC, GLTB2 and rice RBS1 TPs. On the other hand, Vojta et al. (2004) proposed that positively charged amino acid residues at the C-terminus of TPs (positions -8 and -1 from the cleavage site) were involved in atToc34 recognition and import. This seems to be the case for the four Arabidopsis TPs studied here, since they present positively charged residues around the fore-mentioned positions (**Figure 1A**). Contrary, RBS1 TP only presents two positively charged residues at −2 and −4. Our experimental results support that CAB6 and RCA TPs follow the atToc159-dependent import mechanism specific for photosynthetic proteins, what would explain their higher import efficiency into chloroplasts (**Figure 1F**). However, they must also be able to interact with the translocon machinery that is expressed constitutively across all tissues (mainly atToc132, atToc120 and atToc34) in order to be efficiently imported into leucoplasts (**Figure 2D**).

For TOCC and GLTB2 TPs, which show a similar degree of import rate in both plastid types (**Figures 1F**, **2D**), our results support a preferential interaction with the TOC family members uniformly expressed across shoot and root.

The case of rice RBS1 TP is surprising, showing only ability to mediate import to plastids in rice (**Figures 1F**, **2D**, **3D**). This would suggest strong differences between the TIC/TOC systems of the two species analyzed that avoid its recognition by Arabidopsis members. This idea is supported by some previous observations that point to differential behavior of TPs depending on the species they were tested. Pea CAB TP was shown to mediate preferential import of proteins into chloroplast (Wan et al., 1996), while RCA TP conferred no such specificity, exhibiting comparable efficiencies in the import of nuclear-encoded proteins in chloroplasts and leucoplasts (Wan et al., 1995; Wan et al., 1996). As we have shown in this work, in Arabidopsis, both TPs are more effective in their capacity to import eGFP into chloroplasts, although eGFP import into leucoplasts was still quite efficient (**Figure 1F**). On the other hand, all Arabidopsis TPs performed very efficiently in rice chloroplasts (**Figure 3D**), and CAB6, RCA and TOCC abolished completely plastid type specificity in terms of their capacity of protein import (**Figures 4C**, **5A**, **6A**). It seems that all the Arabidopsis TPs in study here lose their plastid type import specificity which is prevalent in their native species environment when expressed in rice cells. Moreover, OsRBS1 TP was completely unable to mediate plastid import in Arabidopsis, suggesting that the mechanisms regulating the import of nuclear-encoded proteins into plastids may not be highly conserved and appear to vary between species.

In general, protoplast transient expression experiments resulted in lower import efficiencies than those observed in plant stable expression. This could be explained by high protein expression being able to saturate the translocon machinery and leading to partial import. Thus, caution should be taken when TPs are characterized using only protoplasts, onion cells or BY-2 cells experiments where these artificial effects are possible.

Our results show low protein accumulation in transgenic plant roots (**Figure 6**) although the promoter used has been reported to drive higher expression in rice roots relative to leaves (Green et al., 2002). Whether this is due to lower protein production or degradation before plastid import remains to be studied. In addition, we note that all lines recovered after transformation with the TOCC TP-eGFP construct showed lower eGFP expression and protein accumulation. Future studies should be aimed at understanding if this can be avoided by any strategy such as specific codon usage optimization for rice.

Targeting of recombinant proteins to plastids has been a difficult task, especially in species where direct plastid genome transformation remains a challenge (Scharff and Bock, 2014). Previous works had shown the failure of TPs to drive recombinant protein import into plastids even when they were used in their same species, like in the case of the 83-amino-acid chloroplast transit peptide of the rice serotonin N-acetyltransferase (SNAT) that was fused to the sheep (*Ovis aries*) SNAT gene and that lead to cytoplasmic protein accumulation in rice (Byeon et al., 2014), highlighting the need for the identification of specific signals that perform correctly in your species of choice. Our work contributes an important tool and the corresponding underpinning biological bases to permit efficient import of nuclear-encoded proteins into plastids. This is important not only for fundamental science investigations but also in terms of biotechnological applications.

## Accession Numbers

Sequence data from this article can be found in the Arabidopsis Information Resource (TAIR) or GenBank under the following accession numbers: CAB6, At3g54890; TOCC, At4g32770; RCA, At2g39730; GLTB2, At2g41220; RECA, At1g79050; and RBS1A, Os12g0274700.

## Data Availability Statement

All datasets presented in this study are included in the article/supplementary material.

## Author Contributions

ÁE, PC, and EC designed the experiments. ÁE conducted protoplast transformation and visualization assays. ÁE and CB performed rice transformation experiments. ÁE, CB, and TC were involved in plant regeneration after callus transformation. CB and VM performed the immune localization of GFP and the electron microscopy in the Servicio Central de Soporte a la Investigación Experimental – Universidad de Valencia. ÁE, PC, LR, and EC wrote the paper.

## Conflict of Interest

The authors declare that the research was conducted in the absence of any commercial or financial relationships that could be construed as a potential conflict of interest.
