# Supplementary material for: Transit Peptides From Photosynthesis-Related Proteins Mediate Import of a Marker Protein Into Different Plastid Types and Within Different Species
Source: Front Plant Sci. 2020 Sep 25;11:560701. doi: 10.3389/fpls.2020.560701 (PMC7545105; doi:10.3389/fpls.2020.560701)
Supplement: Supplementary file 1 [file DataSheet_1.pdf]

Supplementary Data 1. List of modular pieces and primers used.

Modular piece (Level 0)	**Amplification Primers (5' – 3')** (Mutagenesis primers)	Template
*At*CAB6$_{TP}$	5' – ATGAAGACATCTCGGGCTGCTGGTCGTGGCTCGCCAG – 3'<br>5' – ATGAAGACATCTCAAATGGCGTCGAACTCGCTTATG – 3'	Arabidopsis genomic DNA[1]
*At*RCA$_{TP}$	5' – ATGAAGACATCTCGGGCTGCCAACACCTTGAATGATCC – 3'<br>5' – ATGAAGACATCTCAAATGGCCGCCGCAGTTTCCAC – 3'<br>5' – (GCGCGAAGACAAGATAAACAAACCGATGGAGAC) – 3'<br>5' – (GCGCGAAGACTTTATCTTCTTTCACAGCCAACAC) – 3'	Arabidopsis cDNA[1]
*At*TOCC$_{TP}$	5' – ATGAAGACATCTCGGGCTGCCGAAACCCTAGAAATGG – 3'<br>5' – ATGAAGACATCTCAAATGGAGATACGGAGCTTGATTG – 3'	Arabidopsis genomic DNA[1]
*At*RecA$_{TP}$	5' – ATGAAGACATCTCAAATGGATTCACAGCTAGTCTTG – 3'<br>5' – ATGAAGACATCTCGGGCTGCGGCGTAGACGGTAACCGGAG – 3'	Arabidopsis genomic DNA
*At*GLTB2$_{TP}$	5' – ATGAAGACATCTCAAATGGCTCTACAGTCTCCCGGAG – 3'<br>5' – ATGAAGACATCTCGGGCTGCTCGGTCAGAATTAAGGATCG – 3'	Arabidopsis genomic DNA
*Os*Rbs1$_{TP}$	5' – ATGAAGACATCTCAAATGGCCCCCTCCGTGATG– 3'<br>5' – ATGAAGACATCTCGGGCTGCGCACCTGATCCTGCCGCCAT– 3'	*Oryza sativa* subsp. *japonica* genomic DNA
CaMV p35S (short)	5' – ATGAAGACATCTCAGGAGGTCAACATGGTGGAGC – 3'<br>5' – ATGAAGACATCTCGCATTGCGTGTCCTCTCCAAATGA – 3'	pICH41388 (MoClo[2])
CaMV p35S (1,3kb)	5' – ATGAAGACATCTCAGGAGGAATTCCAATCCCACA – 3'<br>5' – ATGAAGACATCTCGGGCTGCGTGTCCTCTCCAAATGA – 3'	pICH51266 (MoClo[2])
2x CaMV p35s + 5'UTR TMV	-	pICH51288 (MoClo[2])
pNOS+Ω	-	pICH87633 (MoClo[2])
pMaize Ubi1+1i	5' – ATGAAGACATCTCAGGAGTGCAGTGCAGCGTGACCCGGTC – 3'<br>5' – ATGAAGACATCTCGCATTCTGCAGAAGTAACACCAAACAA – 3'	Synthesis by GenScript
pMaize Ubi1+1i	5' – ATGAAGACATCTCAGGAGTGCAGTGCAGCGTGACCCGGTC – 3'<br>5' – ATGAAGACATCTCGGGCTGACATTCTGCAGAAGTAACACC – 3'	Synthesis by GenScript
HPT	5' – ATGAAGACATCTCAAATGAAAAAGCCTGAACTCACC – 3'<br>5' – ATGAAGACATCTCGAAGCTATTCCTTTGCCCTCGGAC – 3'	Paul Christou's Lab[3]
eGFP	-	pICH41531 (MoClo[2])
mCherry	-	pICSL50004 (MoClo[2])

p19	-	pICH44022 (MoClo2)
T35s	-	pICH41414 (MoClo2)
tNOS	-	pICH41421 (MoClo2)

1 Eseverri, Á., López-Torrejón, G., Jiang, X., Burén, S., Rubio, L. M., and Caro, E. (2020). Use of synthetic biology tools to optimize the production of active nitrogenase Fe protein in chloroplasts of tobacco leaf cells. *Plant Biotechnol. J.*, pbi.13347. doi:10.1111/pbi.13347.

2 Obtained from the MoClo Plant Parts Kit (Addgene: https://www.addgene.org/cloning/MoClo/patron/)

3 Sudhakar, D., Fu, X., Stoger, E., Williams, S., Spence, J., Brown, D. P., et al. (1998). Expression and immunolocalisation of the snowdrop lectin, GNA in transgenic rice plants. *Transgenic Res.* 7, 371–378. doi:10.1023/A:1008856703464.
