# Supplementary material for: Transit Peptides From Photosynthesis-Related Proteins Mediate Import of a Marker Protein Into Different Plastid Types and Within Different Species
Source: Front Plant Sci. 2020 Sep 25;11:560701. doi: 10.3389/fpls.2020.560701 (PMC7545105; doi:10.3389/fpls.2020.560701)
Supplement: Supplementary file 2 [file DataSheet_2.pdf]

Supplementary Data 2A. List of assembled Transcriptional Units for Arabidopsis experiments.

**Transcriptional Units (Level 1)**	**Entry vector**	**Modular pieces used**
P19	pICH47751	pNOS+Ω::*p19*-tNOS
cyto*eGFP*	pICH47781	p35S::*eGFP*-T35s
*At*RCATP-*eGFP*	pICH47732	2x p35S::*At*RCATP-*eGFP*-tNOS
*At*TOCCTP-*eGFP*	pICH47732	2x p35S::*At*TOCCTP-*eGFP*-tNOS
*At*CAB6TP-*eGFP*	pICH47732	2x p35S::*At*CAB6TP-*eGFP*-tNOS
*At*GLTB2TP-*eGFP*	pICH47732	2x p35S::*At*GLTB2TP-*eGFP*-tNOS
*Os*Rbs1TP-*eGFP*	pICH47732	2x p35S::*Os*Rbs1TP-*eGFP*-tNOS
*At*RecATP-mCherry	pICH47742	2x p35S::*At*RecACTP-mCherry-tNOS

Supplementary Data 2B. List of assembled Transcriptional Units for rice experiments.

**Transcriptional Units (Level 1)**	**Entry vector**	**Modular pieces used**
HPT	pUC57-L1P3	P35s (short)::*HPT*-tNOS
cyto*eGFP*	pUC57-L1P1	pMaize Ubi1 +1::*eGFP*-tNOS
*At*RCATP-*eGFP*	pUC57-L1P1	pMaize Ubi1 + 1::*At*RCATP-*eGFP*-tNOS
*At*TOCCTP-*eGFP*	pUC57-L1P1	pMaize Ubi1 +1::*At*TOCCTP-*eGFP*-tNOS
*At*CAB6TP-*eGFP*	pUC57-L1P1	pMaize Ubi1 +1::*At*CAB6TP-*eGFP*-tNOS
*At*GLTB2TP-*eGFP*	pUC57-L1P1	pMaize Ubi1 +1::*At*GLTB2TP-*eGFP*-tNOS
*Os*Rbs1TP-*eGFP*	pUC57-L1P1	pMaize Ubi1 +1::*Os*Rbs1TP-*eGFP*-tNOS
