# Supplementary material for: Transit Peptides From Photosynthesis-Related Proteins Mediate Import of a Marker Protein Into Different Plastid Types and Within Different Species
Source: Front Plant Sci. 2020 Sep 25;11:560701. doi: 10.3389/fpls.2020.560701 (PMC7545105; doi:10.3389/fpls.2020.560701)
Supplement: Supplementary file 3 [file DataSheet_3.pdf]

Supplementary Data 3. List of multigenic constructions for Arabidopsis experiments.

**Multigenic constructions (Level 2)**	**Entry vector**	**Transcriptional Units used**
cyto*eGFP*+P19	pAGM4673	cyto*eGFP* + P19
*At*RCA$_{TP}$-*eGFP*+P19	pAGM4673	*At*RCA$_{TP}$-*eGFP* + P19
*At*TOCC$_{TP}$-*eGFP*+P19	pAGM4673	*At*TOCC$_{TP}$- *eGFP* + P19
*At*CAB6$_{TP}$-*eGFP*+P19	pAGM4673	*At*CAB6$_{TP}$- *eGFP* + P19
*At*GLTB2$_{TP}$-*eGFP*+P19	pAGM4673	*At*GLTB2$_{TP}$- *eGFP* + P19
*Os*Rbs1$_{TP}$-*eGFP*+P19	pAGM4673	*Os*Rbs1$_{TP}$- *eGFP* + P19
cyto*eGFP* +*At*RecA$_{TP}$-mCherry	pAGM4673	cyto*eGFP* + P19 + *At*RecA$_{TP}$-mCherry
*At*RCA$_{TP}$-*eGFP* +*At*RecA$_{TP}$-mCherry	pAGM4673	*At*RCA$_{TP}$-*eGFP* + P19 + *At*RecA$_{TP}$-mCherry
*At*TOCC$_{TP}$- *eGFP* +*At*RecA$_{TP}$-mCherry	pAGM4673	*At*TOCC$_{TP}$- *eGFP* + P19 + *At*RecA$_{TP}$-mCherry
*At*CAB6$_{TP}$- *eGFP* +*At*RecA$_{TP}$-mCherry	pAGM4673	*At*CAB6$_{TP}$- *eGFP* + P19 + *At*RecA$_{TP}$-mCherry
*At*GLTB2$_{TP}$- *eGFP* +*At*RecA$_{TP}$-mCherry	pAGM4673	*At*GLTB2$_{TP}$- *eGFP* + P19 + *At*RecA$_{TP}$-mCherry
*Os*Rbs1$_{TP}$- *eGFP* +*At*RecA$_{TP}$-mCherry	pAGM4673	*Os*Rbs1$_{TP}$- *eGFP* + P19 + *At*RecA$_{TP}$-mCherry
