# Supplementary material for: Transit Peptides From Photosynthesis-Related Proteins Mediate Import of a Marker Protein Into Different Plastid Types and Within Different Species
Source: Front Plant Sci. 2020 Sep 25;11:560701. doi: 10.3389/fpls.2020.560701 (PMC7545105; doi:10.3389/fpls.2020.560701)
Supplement: Supplementary file 4 [file Image_1.pdf]

Supplementary Figure 1. Gene expression of TOC and TIC family members in *A. thaliana* and *O. sativa*. Data was retrieved from the Plant eFP browser, showing log_2 ratio from microarray transcriptomics of *Arabidopsis thaliana* **(A)** and *Oryza sativa* **(B)** tissues (Waese et al., 2017). **(C)** Homologs of *A. thaliana* TOC and TIC family members in *O. sativa* ssp. japonica. In the case of Toc132-Toc120 and Toc33-Toc34, the same one homolog was retrieved respectively.

(A)

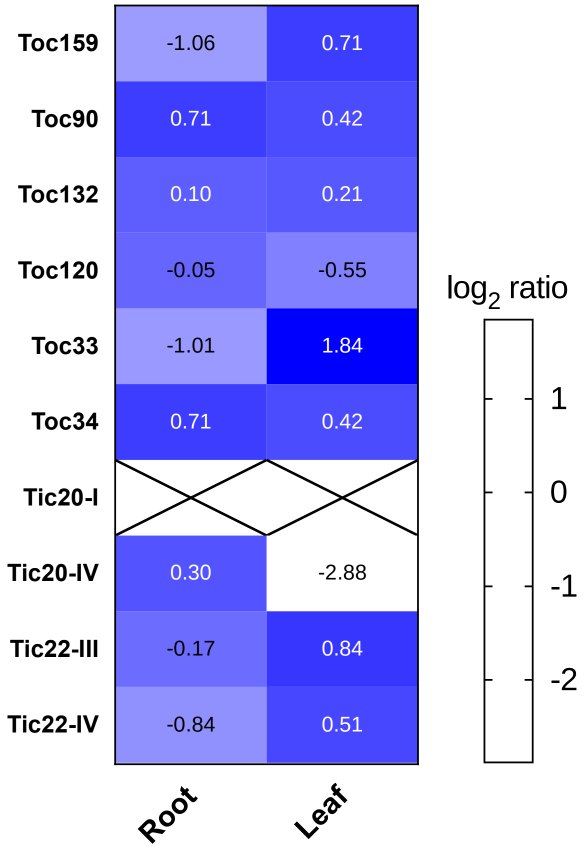

(B)

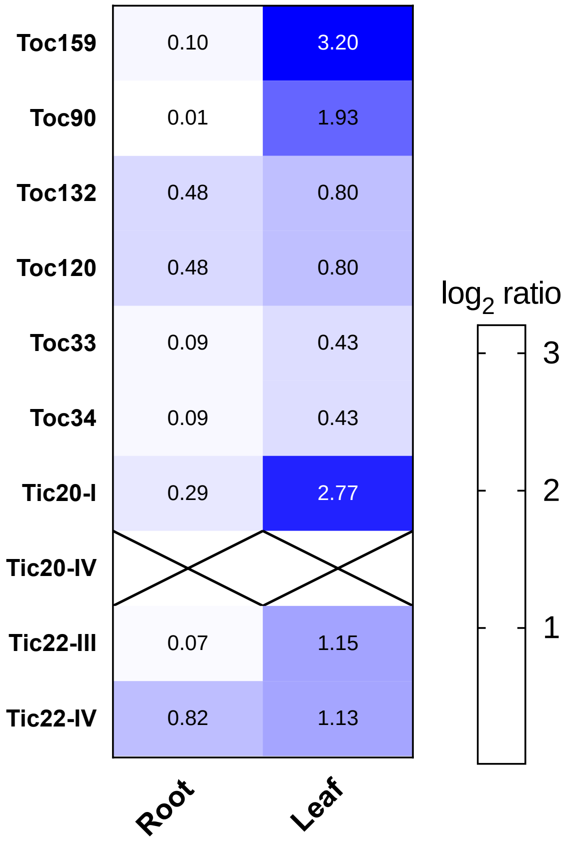

(C)

**Protein**	*Arabidopsis thaliana*	*Oryza sativa*
Toc159	AT4G02510	Os05g0151400
Toc90	AT5G20300	Os12g0197400
Toc132	AT2G16640	Os10g0548800
Toc120	AT3G16620	Os10g0548800
Toc33	AT1G02280	Os03g0240500
Toc34	AT5G05000	Os03g0240500
Tic20-I	AT1G04940	Os07g0568500
Tic20-IV	AT4G03320	-
Tic22-III	AT3G23710	Os07g0290800
Tic22-IV	AT4G33350	Os06g0213200

Bibliography

Waese, J., Fan, J., Pasha, A., Yu, H., Fucile, G., Shi, R., et al. (2017). ePlant: Visualizing and exploring multiple levels of data for hypothesis generation in plant biology. *Plant Cell* 29, 1806–1821. doi:10.1105/tpc.17.00073.
