# Supplementary material for: Transit Peptides From Photosynthesis-Related Proteins Mediate Import of a Marker Protein Into Different Plastid Types and Within Different Species
Source: Front Plant Sci. 2020 Sep 25;11:560701. doi: 10.3389/fpls.2020.560701 (PMC7545105; doi:10.3389/fpls.2020.560701)
Supplement: Supplementary file 5 [file Image_2.pdf]

Supplementary Figure 2. Biological context of *A. thaliana* genes containing selected Transit Peptides. **(A)** Schematic representation of the metabolic pathways in which genes *AT3G54890* (*At*CAB6), *AT2G39730* (*At*RCA), *AT4G32770* (*At*TOCC) and *AT2G41220* (*At*GLTB2) are involved. Illustration obtained from MapMan (Thimm et al., 2004) overview of plant metabolism. **(B)** Gene Ontology Annotations.

(A)

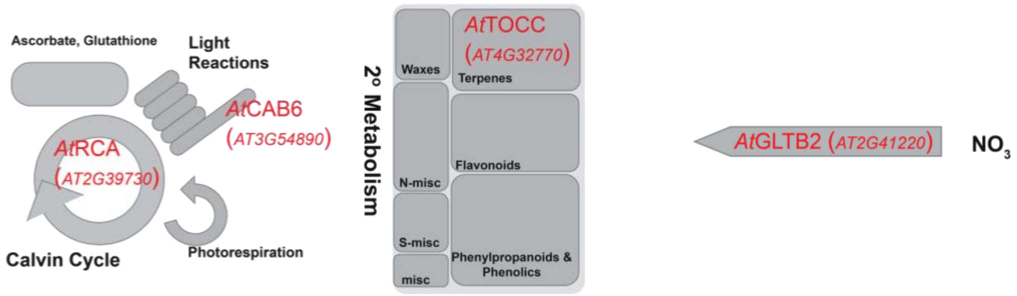

(B)

	***At*CAB6** (*AT3G54890*)	***At*RCA** (*AT2G39730*)	***At*TOCC** (*AT4G32770*)	***At*GLTB2** (*AT2G41220*)
GO Biological Process	Photosynthesis, light harvesting in photosystem I	Response to light stimulus, jasmonic acid	Vitamin E biosynthetic process	Ammonia assimilation cycle, glutamate biosynthetic process
GO Cellular Component	Chloroplast (envelope, thylakoid)	Chloroplast (envelope, stroma, thylakoid)	Chloroplast (envelope, thylakoid)	Chloroplast (envelope, stroma)
GO Molecular Function	Chlorophyll binding	ADP and ATP binding, ribulose-1,5-bisphosphate carboxylase/oxygenase activator activity	Tocopherol cyclase activity	Glutamate synthase (ferredoxin) activity

Bibliography

Thimm, O., Bläsing, O., Gibon, Y., Nagel, A., Meyer, S., Krüger, P., et al. (2004). MAPMAN: A user-driven tool to display genomics data sets onto diagrams of metabolic pathways and other biological processes. *Plant J.* 37, 914–939. doi:10.1111/j.1365-313X.2004.02016.x.
