# Supplementary material for: Transit Peptides From Photosynthesis-Related Proteins Mediate Import of a Marker Protein Into Different Plastid Types and Within Different Species
Source: Front Plant Sci. 2020 Sep 25;11:560701. doi: 10.3389/fpls.2020.560701 (PMC7545105; doi:10.3389/fpls.2020.560701)

Supplementary Figure 3. Isolated protoplasts imaged using light microscopy. Isolated protoplasts from **(A)** *Arabidopsis thaliana* aerial part, **(B)** roots and **(C)** *Oryza sativa* stem and sheath tissue were imaged with a Zeiss AxioPhot Microscope (Zeiss) equipped with a Plan-Neofluar 40X/0.75 objective. Scale bar represents (A) 40 µm or (B-C) 20 µm.

(A)

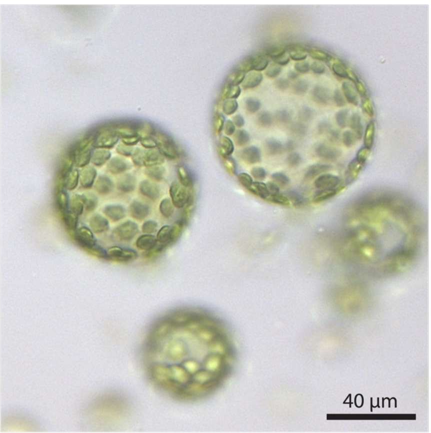

(B)

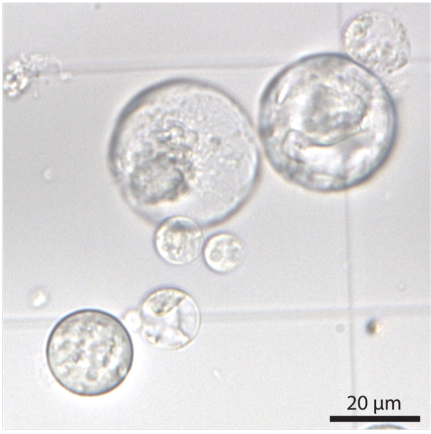

(C)

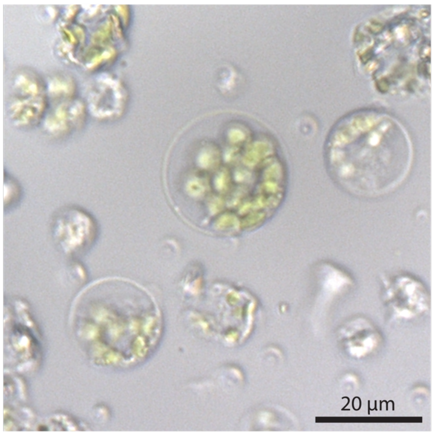

Supplement: Supplementary file 6 [file Image_3.pdf]
