# Supplementary material for: Transit Peptides From Photosynthesis-Related Proteins Mediate Import of a Marker Protein Into Different Plastid Types and Within Different Species
Source: Front Plant Sci. 2020 Sep 25;11:560701. doi: 10.3389/fpls.2020.560701 (PMC7545105; doi:10.3389/fpls.2020.560701)
Supplement: Supplementary file 7 [file Image_4.pdf]

Supplementary Figure 4. Immunogold Transmission Electron Microscopy images treated with polyclonal antibody. Immunogold labeling of eGFP in **(A-D)** proplastids of rice callus cells, **(E-H)** chloroplast of rice leaf cells and **(I-L)** plastids of rice root cells using a GFP polyclonal antibody (diluted 1:250). **A, E, I** Wild-type cells. **B, F, J** *At*CAB6$_{TP}$-eGFP. **C, G, K** *At*RCA$_{TP}$-eGFP. **D, H, L** *At*TOCC$_{TP}$-eGFP. i-vi: Transmission electron microscope (TEM) images. (CW: cell wall, m: mitochondria, N: nucleus, Nu: nucleolus, p: plastid like, St: starch, Ch: chloroplast, p-c: plastid showing an isolated cytoplasmic region; bars = 500 nm; gold particle size = 15 nm)

(A)

WT

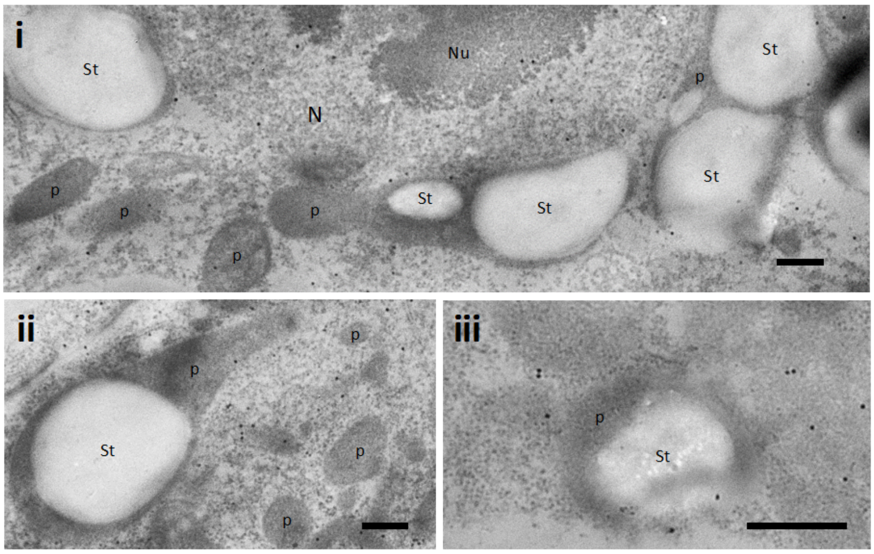

(B)

*At*CAB6$_{TP}$-eGFP

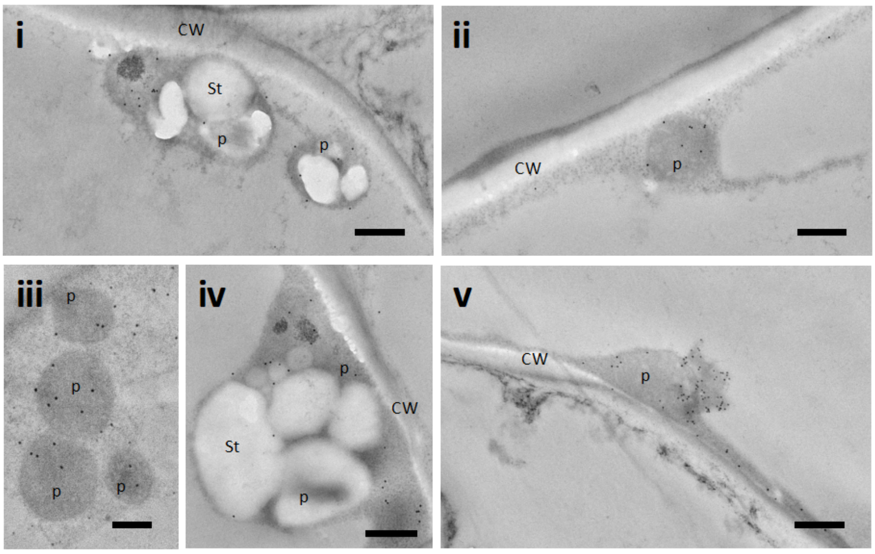

(C)

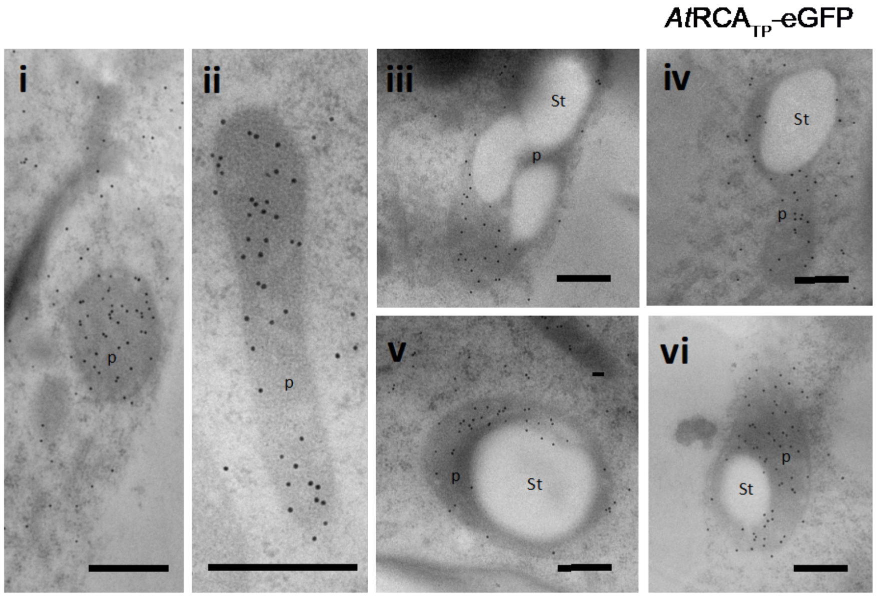

(D)

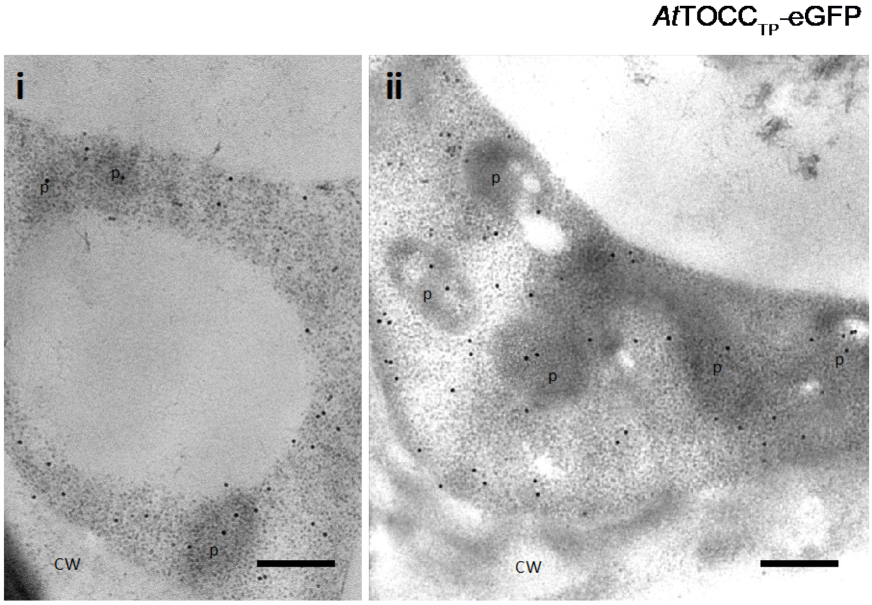

(E)

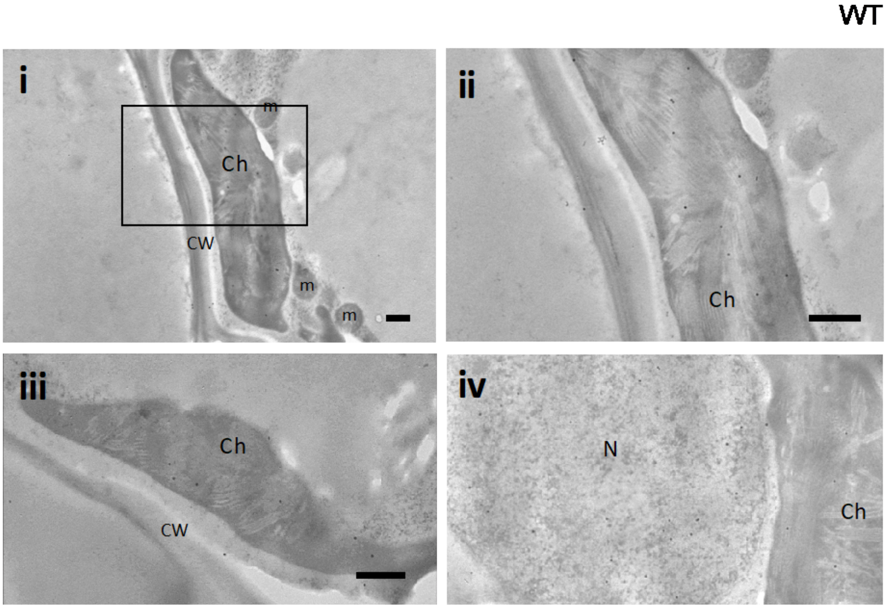
WT
i
m
Ch
CW
m
m
ii
Ch
iii
Ch
CW
iv
N
Ch

(F)

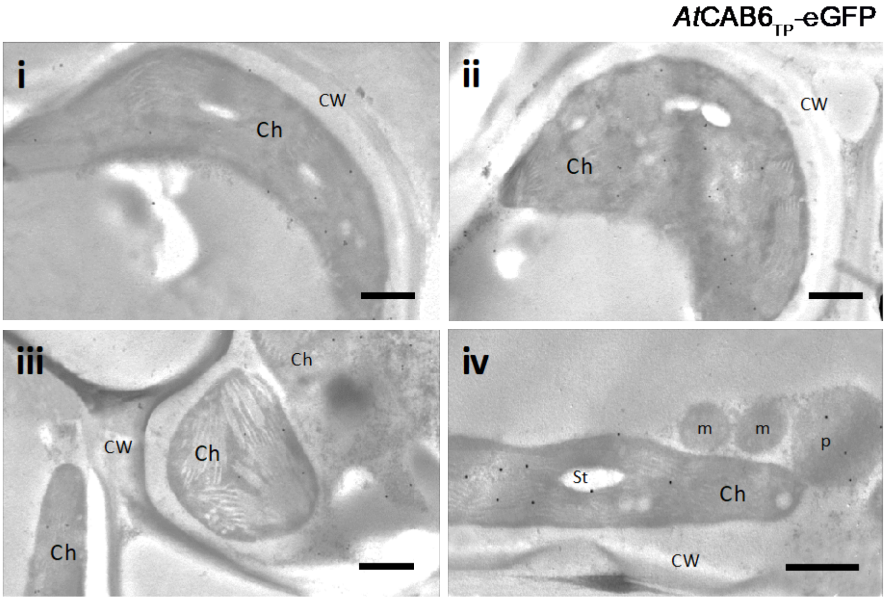
*At*CAB6$_{TP}$-eGFP
i
CW
Ch
ii
CW
Ch
iii
Ch
CW
Ch
Ch
iv
m
m
p
St
Ch
CW

(G)

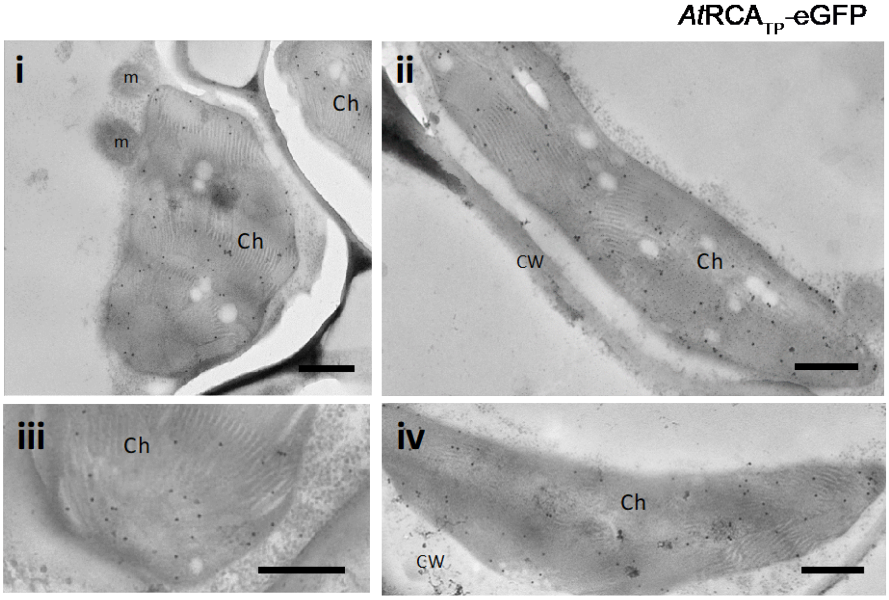
AtRCATP-eGFP
i
m
m
Ch
Ch
ii
CW
Ch
iii
Ch
iv
Ch
CW

(H)

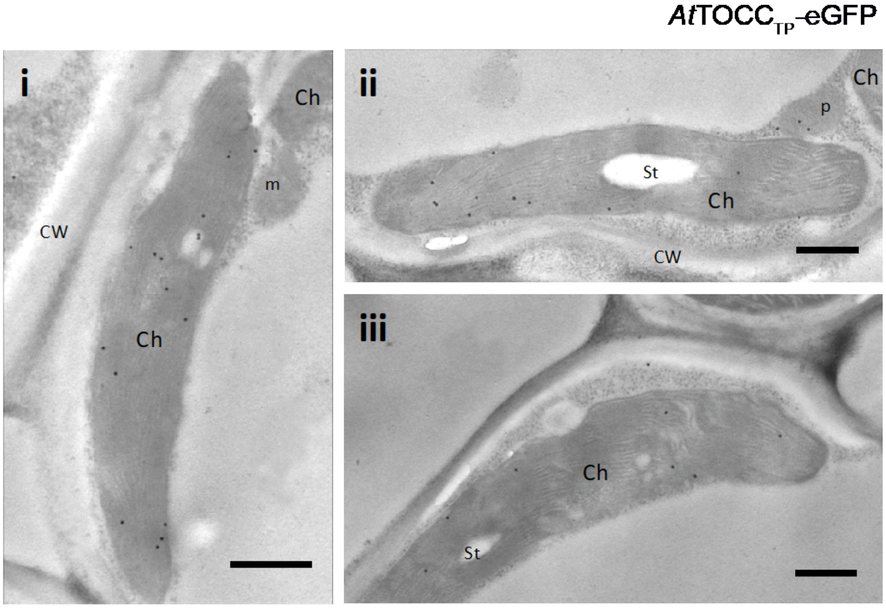
AtTOCCTP-eGFP
i
Ch
m
CW
Ch
ii
Ch
p
St
Ch
CW
iii
Ch
St

(I)

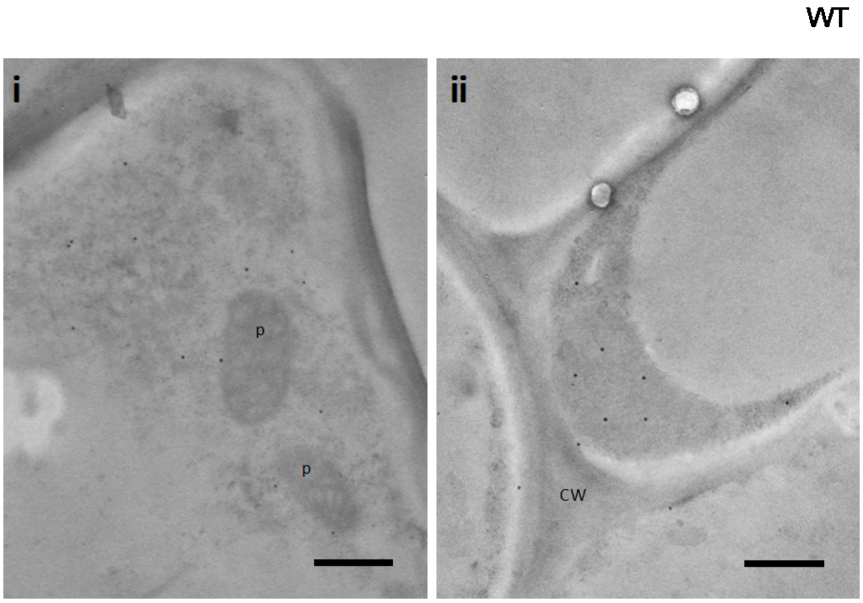

(J)

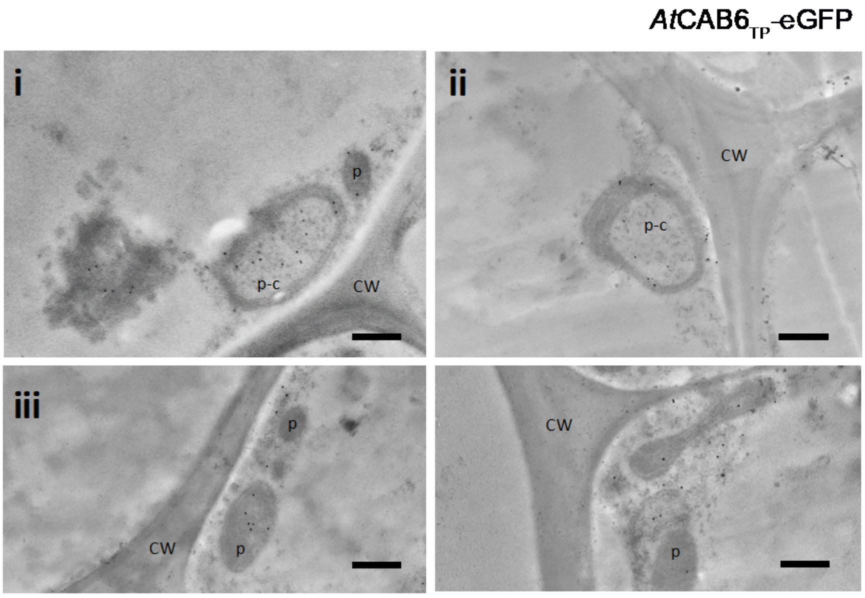

(K)

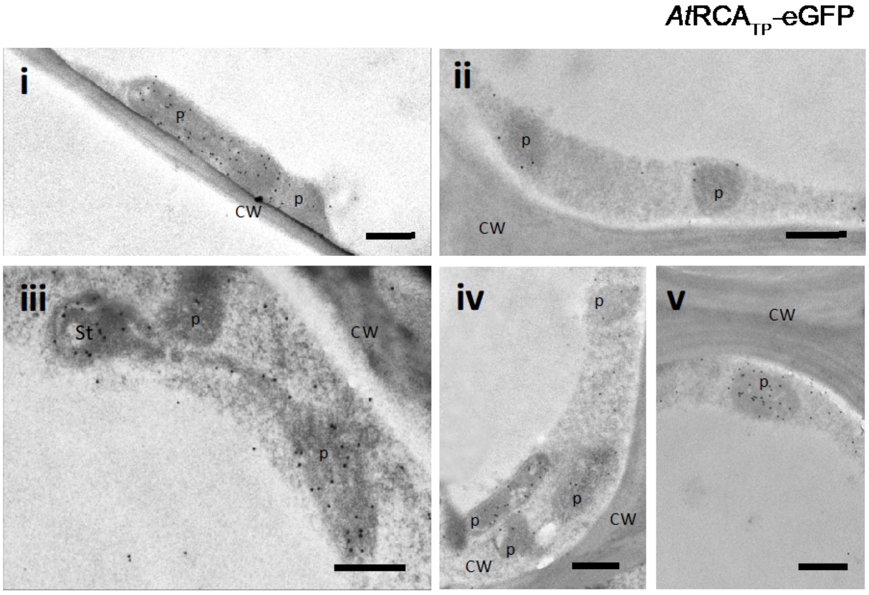
AtRCATP-eGFP
i
p
p
CW
ii
p
p
CW
iii
St
p
CW
p
iv
p
p
p
p
CW
CW
v
CW
p

(L)

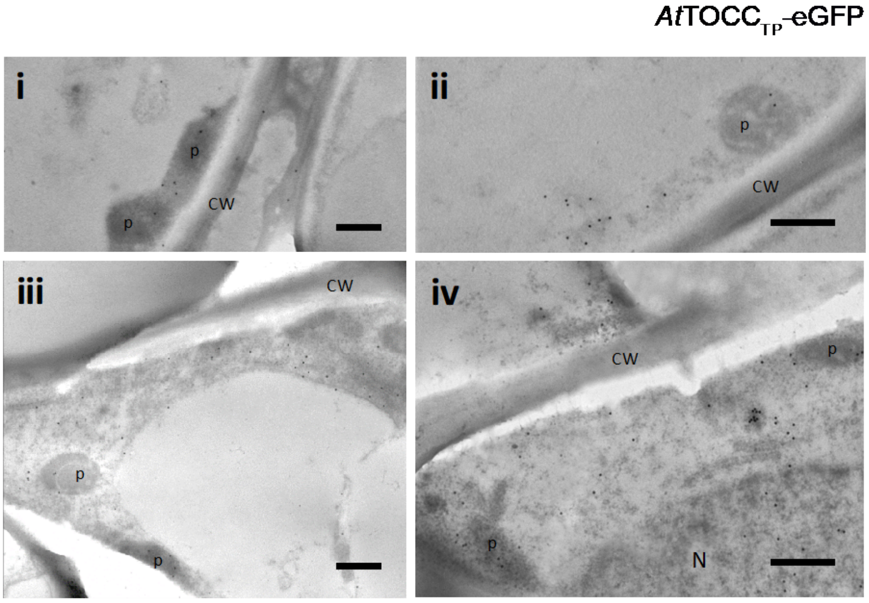
AtTOCCTP-eGFP
i
p
p
CW
ii
p
CW
iii
CW
p
p
iv
CW
p
p
N
