# Supplementary material for: Transit Peptides From Photosynthesis-Related Proteins Mediate Import of a Marker Protein Into Different Plastid Types and Within Different Species
Source: Front Plant Sci. 2020 Sep 25;11:560701. doi: 10.3389/fpls.2020.560701 (PMC7545105; doi:10.3389/fpls.2020.560701)

Supplementary Figure 5. Confocal laser scanning microscopy images of wild-type and transgenic lines rice leaves. Confocal laser scanning microscopy images of wild- type (WT) rice leaves and leaves from transgenic lines transformed with *At*CAB6TP-eGFP, *At*RCATP-eGFP and *At*TOCCTP-eGFP fusions. The four panels show the individual signals for eGFP (a) chlorophyll autofluorescence (b), transmitted light (c) and the overlap of eGFP and chlorophyll autofluorescence (d). Scale bars, 20 µm.

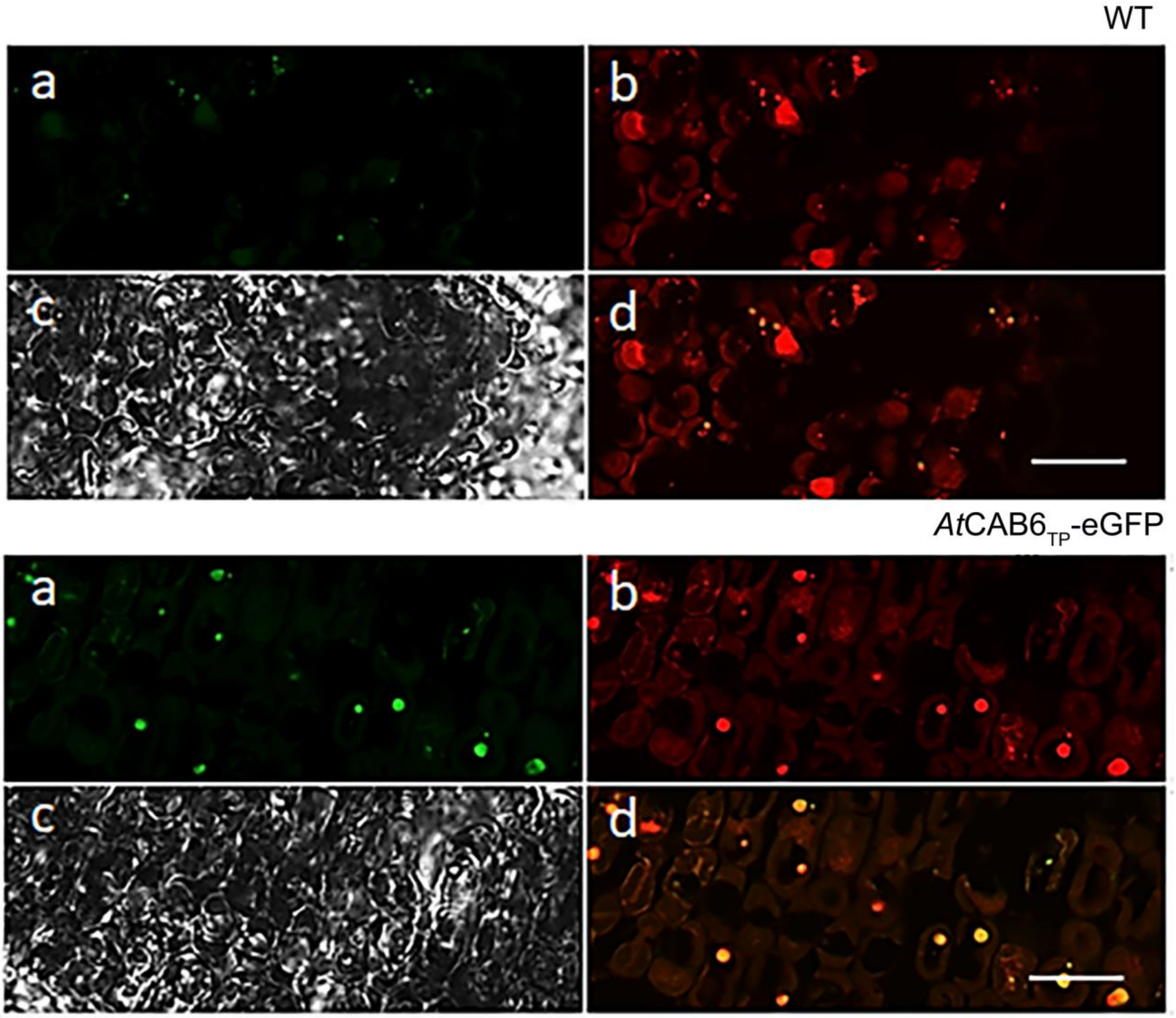

At$TOCC_{TP}$-eGFP

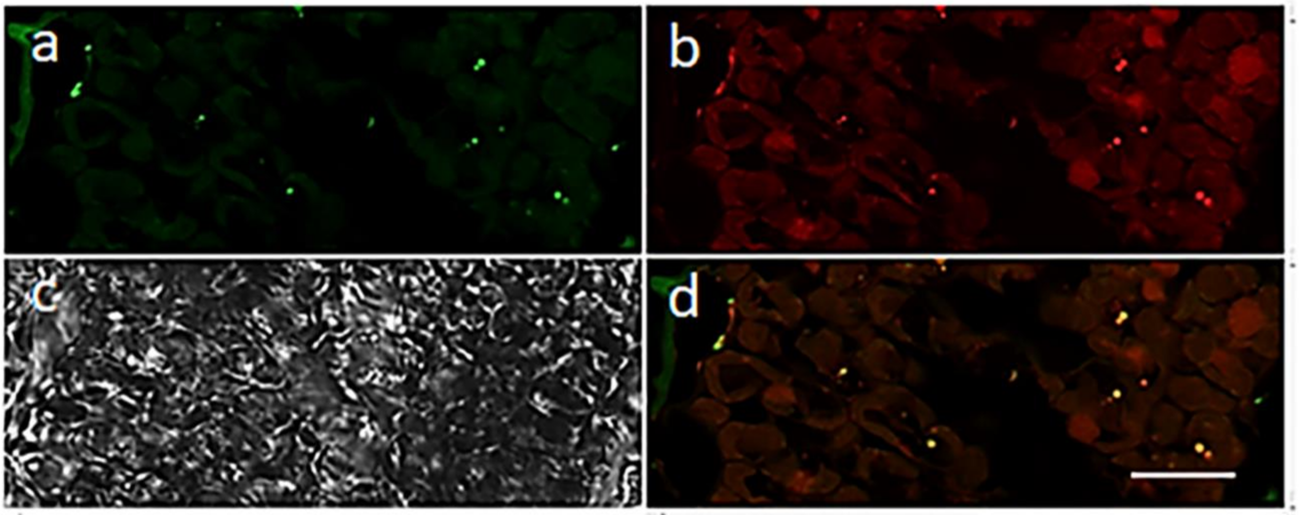

AtRCA_{TP}-eGFP

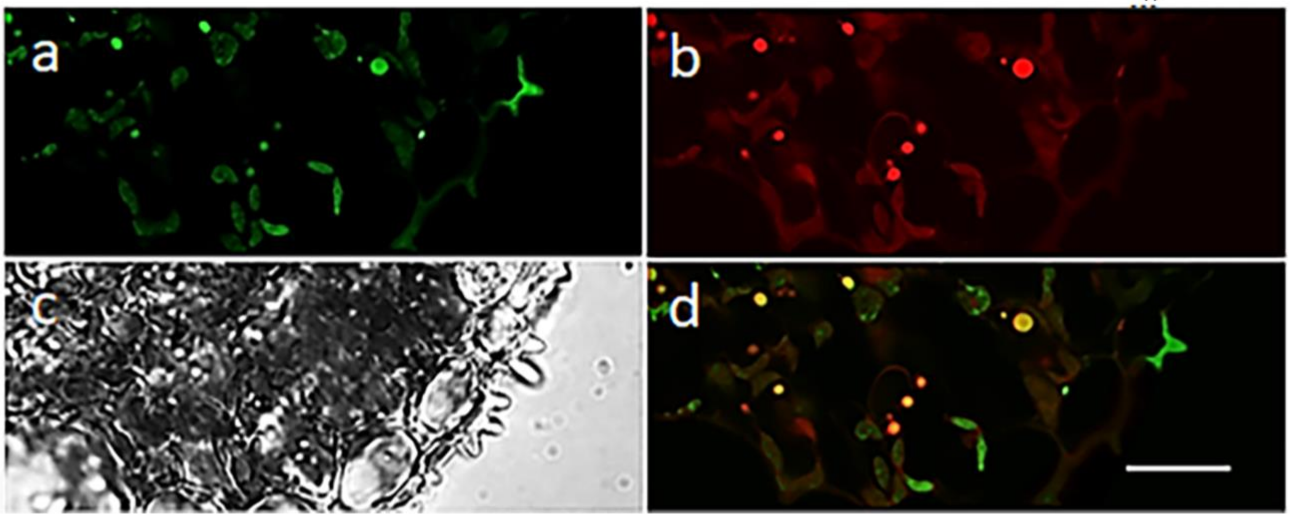

Supplement: Supplementary file 8 [file Image_5.pdf]
