# Supplementary material for: Transit Peptides From Photosynthesis-Related Proteins Mediate Import of a Marker Protein Into Different Plastid Types and Within Different Species
Source: Front Plant Sci. 2020 Sep 25;11:560701. doi: 10.3389/fpls.2020.560701 (PMC7545105; doi:10.3389/fpls.2020.560701)

Supplementary Figure 6. Characteristics of *A. thaliana* and *O. sativa* Transit Peptides. Relative abundance of Alanine, Serine and uncharged amino acids (Serine, Cysteine, Threonine, Tyrosine, Asparagine, Glutamine) in the minimal TPs and the mature protein.

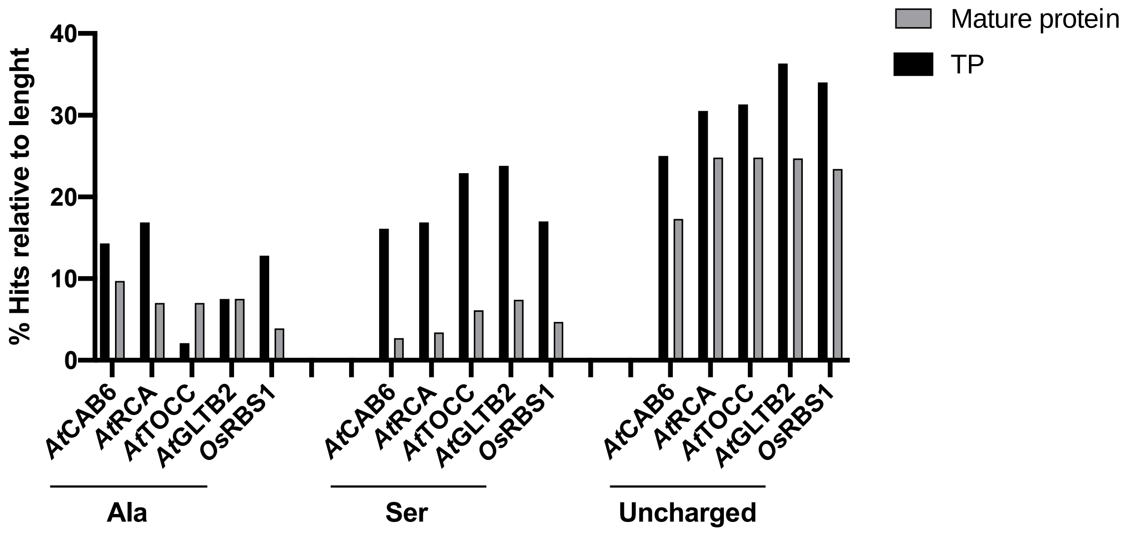

Supplement: Supplementary file 9 [file Image_6.pdf]
