# Supplementary material for: Transit Peptides From Photosynthesis-Related Proteins Mediate Import of a Marker Protein Into Different Plastid Types and Within Different Species
Source: Front Plant Sci. 2020 Sep 25;11:560701. doi: 10.3389/fpls.2020.560701 (PMC7545105; doi:10.3389/fpls.2020.560701)
Supplement: Supplementary file 10 [file Table_1.pdf]

Supplementary Table 1. Statistics of import efficiency of TPs. **(A)** Import efficiency of TPs into Arabidopsis chloroplast and root plastids, and rice chloroplast in transient expression protoplast experiments. **(B)** Import efficiency of TPs into rice proplastids, chloroplast and root plastids. Import efficiency was defined as the percentage of the processed faster-migrating protein form relative to the total amount of expressed eGFP protein. Data represent means (n=3) with SD, except GLTB2 and RBS1 TPs tested in root plastids, where n=2. **(C-E)** Multiple Student's test between tissues in protoplast transient experiments at (C) 12 hours and (E) 16h post-transfection and (D) stable transgenic rice lines. Student's test significant differences ($P < 0.05$).

(A)

	**Arabidopsis chloroplast**			**Arabidopsis leucoplast**			**Rice chloroplast**		
Protoplast	12 h	16 h	N	12 h	16 h	N	12 h	16 h	N
*At*CAB6TP	95,82 ± 1,94	95,20 ± 0,96	3	60,57 ± 1,36	71,24 ± 9,51	3	80,95 ± 4,03	81,06 ± 2,98	3
*At*RCATP	92,32 ± 5,66	94,32 ± 4,88	3	56,16 ± 3,34	53,28 ± 0,75	3	85,60 ± 4,06	88,50 ± 2,16	3
*At*TOCCTP	71,41 ± 15,94	63,09 ± 17,67	3	50,80 ± 3,53	52,01 ± 8,79	3	89,87 ± 0,44	92,10 ± 1,30	3
*At*GLBT2TP	56,87 ± 2,66	79,46 ± 7,93	3	50,78 ± 1,99	49,18 ± 3,95	2	97,64 ± 1,92	99,07 ± 0,68	3
*Os*RBS1TP	2,57 ± 1,74	2,39 ± 0,76	3	3,28 ± 3,36	2,29 ± 1,88	2	68,38 ± 0,53	68,24 ± 4,47	3

(B)

Stable rice lines	**Callus proplastids**		**Leaf chloroplast**		**Root leucoplast**	
	% ± SD	N	% ± SD	N	% ± SD	N
*At*CAB6TP	99,41 ± 0,78	3	98,64 ± 0,98	3	99,93 ± 0,07	3
*At*RCATP	99,55 ± 0,16	3	98,03 ± 1,46	3	99,46 ± 0,09	3
*At*TOCCTP	97,83 ± 1,39	3	99,66 ± 0,28	3	99,68 ± 0,19	3

(C)

12 hours	**Arabidopsis chloroplast**	**Arabidopsis leucoplast**	**p-value**	**Arabidopsis chloroplast**	**Rice chloroplast**	**p-value**
*At*CAB6$_{TP}$	95,82	60,57	0,000014	95,82	80,95	0,004532
*At*RCA$_{TP}$	92,32	56,16	0,000676	92,32	85,60	0,169540
*At*TOCC$_{TP}$	71,41	50,80	0,094104	71,41	89,87	0,115386
*At*GLBT2$_{TP}$	56,87	50,78	0,072910	56,87	97,64	0,000027
*Os*RBS1$_{TP}$	2,568	3,280	0,766732	2,568	68,38	<0,000001

(D)

16 hours	**Arabidopsis chloroplast**	**Arabidopsis leucoplast**	**p-value**	**Arabidopsis chloroplast**	**Rice chloroplast**	**p-value**
*At*CAB6$_{TP}$	95,20	71,24	0,012263	95,20	81,06	0,001446
*At*RCA$_{TP}$	94,32	53,28	0,000135	94,32	88,50	0,131998
*At*TOCC$_{TP}$	63,09	52,01	0,386146	63,09	92,10	0,047101
*At*GLBT2$_{TP}$	79,46	49,18	0,016899	79,46	99,07	0,012967
*Os*RBS1$_{TP}$	2,390	2,288	0,934972	2,390	68,24	0,000015

(E)

Stable rice lines	**Callus proplastids**	**Leaf chloroplast**	**p-value**	**Callus proplastids**	**Root leucoplast**	**p-value**	**Leaf chloroplast**	**Root leucoplast**	**p-value**
*At*CAB6$_{TP}$	99,41	98,64	0,345617	99,41	99,93	0,313229	98,64	99,93	0,085177
*At*RCA$_{TP}$	99,55	98,03	0,147788	99,55	99,46	0,443386	98,03	99,46	0,165593
*At*TOCC$_{TP}$	97,83	99,66	0,088105	97,83	99,68	0,084426	99,66	99,68	0,949813
