# Supplementary material for: Transit Peptides From Photosynthesis-Related Proteins Mediate Import of a Marker Protein Into Different Plastid Types and Within Different Species
Source: Front Plant Sci. 2020 Sep 25;11:560701. doi: 10.3389/fpls.2020.560701 (PMC7545105; doi:10.3389/fpls.2020.560701)
Supplement: Supplementary file 11 [file Table_2.pdf]

Supplementary Table 2. Levels of immunogold labeling. Number of gold particles observed on organelles and cytoplasm of cells of different samples and lines after using a monoclonal or polyclonal antiserum, on a scale indicating: - (not labelling), +/- (very light labelling), + (weak but specific labelling), ++ (specific labelling), +++ (more specific labelling) and ++++++ (specific and strong labelling).

Antiserum: Monoclonal		**WT**	***At*CAB6$_{TP}$-eGFP**	***At*RCA$_{TP}$-eGFP**	***At*TOCC$_{TP}$-eGFP**
**Callus**	Plastids	-	+	++	+
	Mitochondria	-	-	-	-
	Nuclei/nucleoli	-	-	-	-
	Cytoplasm	-	-	-	-
**Leaf**	Chloroplasts	-	+	+++	+
	Mitochondria	-	-	-	-
	Nuclei/nucleoli	-	-	-	-
	Cytoplasm	-	-	-	-
**Root**	Plastids	-	+	++	+
	Mitochondria	-	-	-	-
	Nuclei/nucleoli	-	-	-	-
	Cytoplasm	-	-	-	-
Antiserum: Polyclonal		**WT**	***At*CAB6$_{TP}$-eGFP**	***At*RCA$_{TP}$-eGFP**	***At*TOCC$_{TP}$-eGFP**
**Callus**	Plastids	-	+++	++++++	++
	Mitochondria	-	-	-	-
	Nuclei/nucleoli	+	+	+	+
	Cytoplasm	+/-	+/-	+/-	+/-
**Leaf**	Chloroplasts	+/-	+++	++++++	++
	Mitochondria	-	-	-	-
	Nuclei/nucleoli	+	+	+	+
	Cytoplasm	+/-	+/-	+/-	+/-
**Root**	Plastids	-	++	++++++	++
	Mitochondria	-	-	-	-
	Nuclei/nucleoli	+	+	+	+
	Cytoplasm	+/-	+/-	+/-	+/-
